# The surfaceome of multiple myeloma cells suggests potential immunotherapeutic strategies and protein markers of drug resistance

**DOI:** 10.1038/s41467-022-31810-6

**Published:** 2022-07-15

**Authors:** Ian D. Ferguson, Bonell Patiño-Escobar, Sami T. Tuomivaara, Yu-Hsiu T. Lin, Matthew A. Nix, Kevin K. Leung, Corynn Kasap, Emilio Ramos, Wilson Nieves Vasquez, Alexis Talbot, Martina Hale, Akul Naik, Audrey Kishishita, Priya Choudhry, Antonia Lopez-Girona, Weili Miao, Sandy W. Wong, Jeffrey L. Wolf, Thomas G. Martin, Nina Shah, Scott Vandenberg, Sonam Prakash, Lenka Besse, Christoph Driessen, Avery D. Posey, R. Dyche Mullins, Justin Eyquem, James A. Wells, Arun P. Wiita

**Affiliations:** 1 Department of Laboratory Medicine, University of California, San Francisco, CA USA; 2 Department of Pharmaceutical Chemistry, University of California, San Francisco, CA USA; 3 Department of Medicine, Division of Hematology/Oncology, University of California, San Francisco, CA USA; 4 Department of Cellular and Molecular Pharmacology, University of California, San Francisco, CA USA; 5 INSERM U976, Institut de Recherche Saint Louis, Université de Paris, Paris, France; 6 Program in Chemistry and Chemical Biology, University of California, San Francisco, CA USA; 7 Bristol Myers Squibb/Celgene, San Diego, CA USA; 8 Program in Epithelial Biology, Stanford University School of Medicine, Stanford, CA USA; 9 Department of Pathology, University of California, San Francisco, CA USA; 10 Department of Medical Oncology and Hematology, Kantonsspital St. Gallen, St. Gallen, Switzerland; 11 Department of Systems Pharmacology and Translational Therapeutics, University of Pennsylvania School of Medicine, Philadelphia, PA USA; 12 Corporal Michael J. Crescenz VA Medical Center, Philadelphia, PA USA; 13 Howard Hughes Medical Institute, San Francisco, CA USA; 14 Parker Institute for Cancer Immunotherapy, San Francisco, CA USA; 15 Gladstone Institute for Genomic Immunology, San Francisco, CA USA; 16 Present Address: Cancer Biology Program, Stanford University School of Medicine, Stanford, CA USA

**Keywords:** Mass spectrometry, Myeloma, Proteomics, Membrane proteins

## Abstract

The myeloma surface proteome (surfaceome) determines tumor interaction with the microenvironment and serves as an emerging arena for therapeutic development. Here, we use glycoprotein capture proteomics to define the myeloma surfaceome at baseline, in drug resistance, and in response to acute drug treatment. We provide a scoring system for surface antigens and identify CCR10 as a promising target in this disease expressed widely on malignant plasma cells. We engineer proof-of-principle chimeric antigen receptor (CAR) T-cells targeting CCR10 using its natural ligand CCL27. In myeloma models we identify proteins that could serve as markers of resistance to bortezomib and lenalidomide, including CD53, CD10, EVI2B, and CD33. We find that acute lenalidomide treatment increases activity of MUC1-targeting CAR-T cells through antigen upregulation. Finally, we develop a miniaturized surface proteomic protocol for profiling primary plasma cell samples with low inputs. These approaches and datasets may contribute to the biological, therapeutic, and diagnostic understanding of myeloma.

## Abstract (web summary)

The myeloma cell surface proteome regulates plasma cell biology and delineates therapy targets. Here, the authors profile the myeloma surfaceome at baseline and in drug resistance, finding the potential target CCR10, and include a streamlined approach to primary sample analysis.

## Introduction

The composition of the tumor cell surface plays a central role in determining cancer’s interaction with the local microenvironment. Over the past several years, targeting tumor surface proteins has also rapidly emerged as one of the most exciting frontiers for treating cancer. This strategy is particularly relevant in the case of the plasma cell malignancy multiple myeloma. Antibody-based therapeutics targeting CD38 and SLAMF7 as well as a cellular therapy targeting BCMA have now been FDA-approved. Furthermore, identifying and quantifying cell surface markers play a critical role in the diagnosis and monitoring of all hematologic malignancies, including myeloma.

One of the notable clinical features of myeloma is that despite the recent advent of many effective therapies, there is still no known cure for this disease. Resistance to current small molecule therapeutics, particularly proteasome inhibitors (PIs) such as bortezomib (Btz) and carfilzomib (Cfz), and immunomodulatory drugs such as lenalidomide (Len), is a widespread conundrum. Characterizing surface proteomic changes in these contexts may reveal new strategies to diagnose and specifically treat drug-resistant disease.

Despite the importance of the cell surface to diagnosis, therapy, and biology of myeloma, much remains unknown. While extensive RNA-seq datasets are available on both myeloma primary samples (such as the Multiple Myeloma Research Foundation CoMMpass study (https://research.themmrf.org)) and cell lines (https://www.keatslab.org/data-repository, and Cancer Cell Line Encyclopedia^1^), it is well-known that transcript-level expression is only modestly correlated with surface protein expression^2–4^. This lack of predictive power is related to two main features. First, even for proteins exclusively present at the plasma membrane, transcript-only quantification cannot capture alterations in translational regulation and protein trafficking that ultimately govern surface expression. Second, proteins expressed at the cell surface can also have significant pools of either intracellular or secreted forms as well; RNA-seq cannot distinguish these components. Therefore, these datasets can at best be considered partially predictive of the cell surface proteome.

Other studies have used flow cytometry, or, more recently, mass cytometry/CyTOF^5^, to profile myeloma tumor cells in the context of inter-patient heterogeneity^6^, response to therapy^7^, or drug resistance^8^. However, these assays are typically restricted to monitoring a maximum of ~50 surface proteins that are already very well-characterized and have high-quality antibodies available. Even a recent large-scale survey of normal human B-cells by CyTOF, screening an exhaustive catalog of 351 metal-conjugated antibodies, only identified 98 expressed surface proteins^9^. It is estimated that most human cells express 500–1000 unique proteins on their surface^10^. Therefore, these widely-used approaches can only begin to outline the overall profile of the myeloma cell surface.

To overcome these limitations, here we use a relatively unbiased approach, glycoprotein cell surface capture (CSC)^11^, to directly quantify hundreds of proteins localized to the surface of myeloma tumor cells. We specifically identify potential immunotherapy strategies in myeloma and biomarker candidates of small molecule resistance and response to acute treatment. Finally, to streamline the surface proteomics methodology we develop a miniaturized CSC approach and apply this approach to primary patient myeloma. Uncovering the surface landscape of malignant plasma cells serves as a resource to the myeloma community.

## Results

### Determining the malignant plasma cell surface landscape

**Fig. 1:**
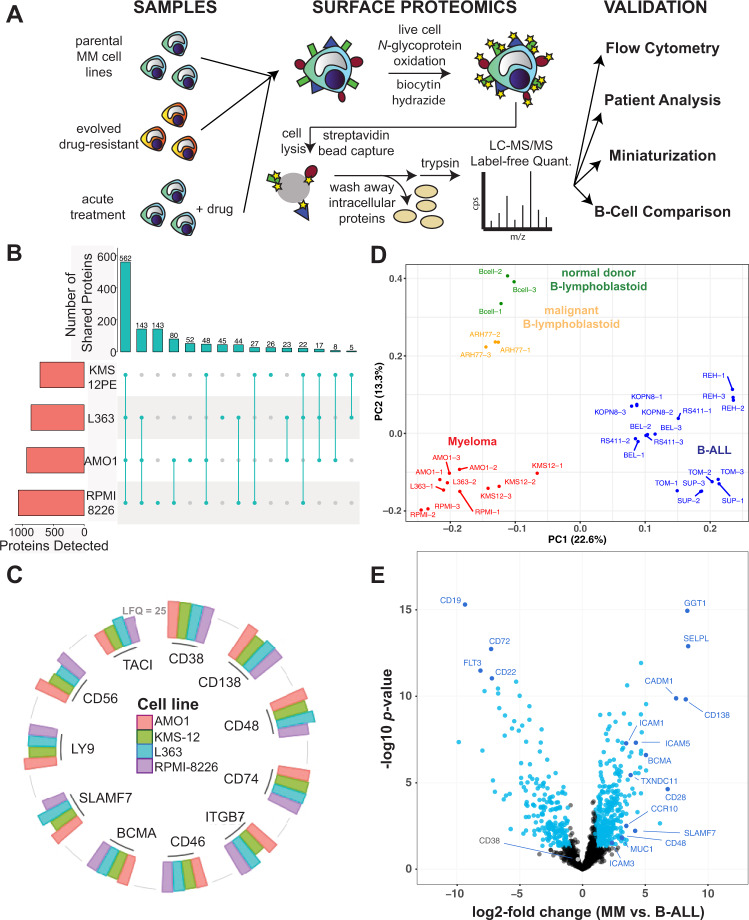
Initial elucidation of the myeloma plasma cell surfaceome. **A** Overall schematic of surface proteomic investigations in this study. This includes a description of the modified cell surface capture (CSC) methodology used, with biotinylated proteins identified after on-bead trypsinization. **B** Upset plot shows high degree of overlap in identified glycoproteins, filtered for annotated membrane proteins, across the four evaluated myeloma cell lines. Data included if identified with two peptides in at least one of three biological replicates per cell line. **C** Common myeloma diagnostic markers and immunotherapeutic targets were identified by cell surface proteomics in all four evaluated cell lines. Height of column indicates label-free quantification (LFQ) intensity from MaxQuant, averaged across biological replicate samples (AMO1: *n* = 3, L363: *n* = 2, KMS12: *n* = 3, RPMI-8226: *n* = 3). A threshold of LFQ = 25 is indicated by gray line. **D** Principal component analysis (PCA) illustrates the differential cell surface landscape of myeloma cells versus B-lymphoblastoid cells and B-cell acute lymphoblastic leukemia cell lines. **E** Volcano plot comparing glycoprotein LFQ intensity of four myeloma cell lines (replicate information listed above for **C**) to eight B-ALL cell lines (*n* = 3 biological replicates each). Significantly changed proteins colored in blue (log_2_-fold change > |1 | ; *p* < 0.05 by *t*-test). For **B**–**E**, source data in Supplementary Data 1.

We first utilized the CSC approach to oxidize, covalently biotinylate, and then isolate N-linked glycoproteins from four multiple myeloma cell lines (KMS-12PE, AMO1, RPMI-8226, L363) (Fig. 1A). Using the label-free quantification approach in MaxQuant^12^, we cumulatively quantified 1245 proteins annotated as membrane bound in Uniprot across all cell lines (range 715–1069 per cell line; minimum two peptides per protein), with a common intersection of 562 proteins (Fig. 1B). To maximize the number of captured proteins, we used glycoprotein biotinylation with on-bead trypsinization (Fig. 1A); however, this method may also spuriously elute peptides deriving from background intracellular proteins or cell surface proteins localized intracellularly. Filtering our data with a recently-described set of the best-validated plasma membrane proteins^10^, 530 of these quantified proteins (305–436 per cell line) appear localized to the cell surface with high confidence (Supplementary Fig. 1A). Notably, across these lines we detected almost all of the major immunotherapy targets in myeloma, as well as canonical flow cytometry markers for plasma cells^7,13^: BCMA, CD138/SDC1, CD38, CD56, SLAMF7/CS-1, CD46, Integrin-b7 (ITGB7), CD74/HLA-DR, TACI, CD48/SLAMF2, and LY9/CD229 (Fig. 1C; Supplementary Data 1).

As myeloma is a malignancy of plasma cells, we were curious what surface markers particularly distinguish myeloma from B-cells earlier in the developmental trajectory. We therefore compared our myeloma surfaceome to our earlier dataset^14^ including six B-cell acute lymphoblastic leukemia (B-ALL) lines derived from early (pre- or pro-) B-cell developmental stages. We further compared the myeloma surface profile to two B-lymphoblastoid cell lines (EBV-immortalized normal cord blood donor B-cells and ARH-77), derived from differentiated, late-stage B-cells^15^. By Principal Component Analysis (PCA) we were encouraged to find that lines representing each cell type (plasma cells, early B-cell, late B-cell) clustered separately, consistent with a relatively unique surface signature for each cell type (Fig. 1D).

We next identified specific markers that most distinguish myeloma plasma cells versus B-cell types (Fig. 1D, E; Supplementary Fig. 1C). Giving confidence in this analysis, many canonical markers were among the strongest hits including BCMA, CD138/SDC1, and CD28 for plasma cells and CD19, CD22, and CD72 for B-ALL cells, respectively (Fig. 1E). Comparison of myeloma cells to the two B-lymphoblastoid cell lines also showed CD138/SDC1 and CD28 as characterizing myeloma cells, while CD19, CD22, and CD20 characterized late-stage B-cells. We were surprised to find the three proteins that most-distinguished myeloma plasma cells from B-ALL, based on fold-change and *p*-value, were not canonical markers at all: gamma-glutamyl transferase 1 (*GGT1*), selectin-P ligand (*SELPLG*), and cell adhesion molecule 1 (*CADM1*) (Fig. 1E). These proteins may carry previously unexplored relevance to myeloma or broader plasma cell biology.

In parallel with our surface proteomic data, we also obtained RNA-seq data for all analyzed cell lines. We overall found a moderate quantitative correlation (Pearson *R* = 0.54; *p* < 2.2e − 16) between the transcriptome and surface proteome of myeloma and B-ALL cells (Supplementary Fig. 1D), consistent with prior studies^14,16^. We did find a set of proteins, including CD99, GGT1, and TOR1AIP2, with transcript level discordant with surface protein, suggestive of possible post-transcriptional regulation (Supplementary Fig. 1D). Taken together, this initial profiling underscores the unique surface phenotype of malignant plasma cells and unexpected markers distinguishing them from closely related cellular models.

### Identifying targets for myeloma antigen-specific immunotherapies

**Fig. 2:**
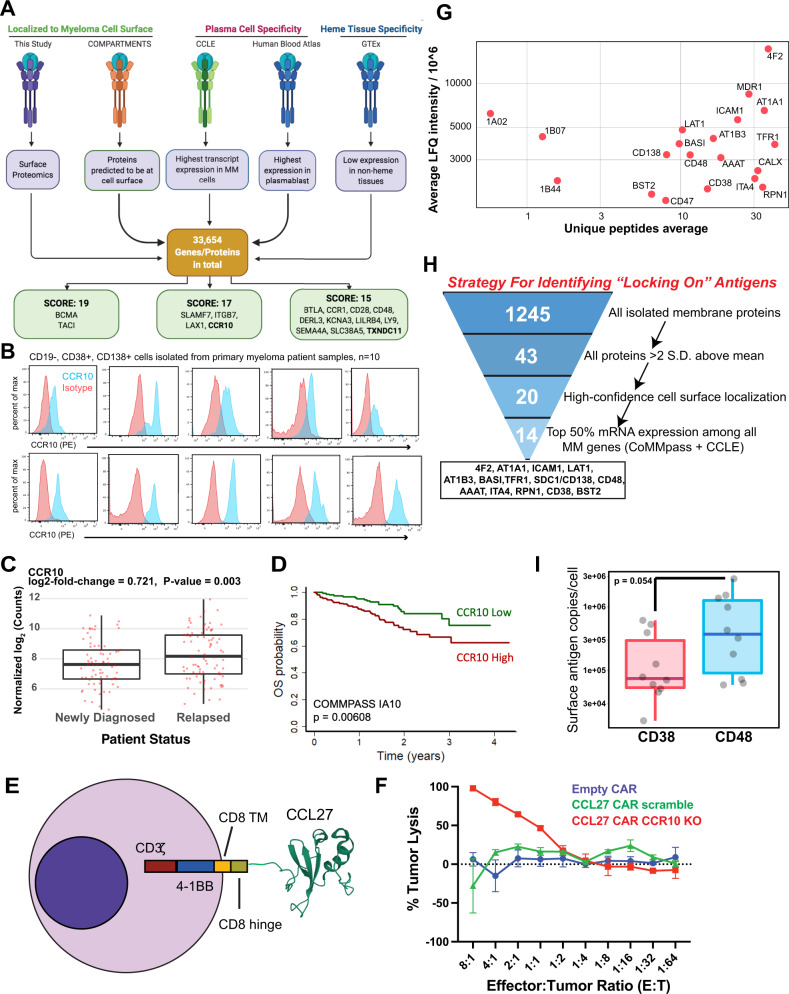
Immunotherapeutically targeting the myeloma cell surfaceome. **A** Outline of a five-criteria scoring strategy, integrating surface proteomics data here with publicly-available mRNA transcriptome data, to propose new targets for possible antigen-specific immunotherapies in myeloma (see “Methods” for details). We specifically point out surface proteins with the highest scores among the total 33,654 analyzed (see Supplementary Table 1 for scoring rubric; maximum score = 19). **B** CCR10 expression measured by flow cytometry in CD19−/CD38+/CD138+ cells isolated from primary myeloma patient samples (*n* = 10 patients). **C** *CCR10* RNA levels for patients in CoMMpass myeloma dataset (release = IA19) separated into newly diagnosed or relapsed groups (*n* = 162). *p*-value from two-sided *t*-test. **D** Overall survival in CoMMpass dataset stratified by CCR10 level. High and Low represent top and bottom 25% of patients by CCR10 gene expression, respectively. Number of patients represented in survival plot is 322. **E** Schematic for CCL27-CAR, including the CD8 hinge and transmembrane domain (TM), 4-1BB co-stimulatory domain, and CD3ζ signaling domain. **F** Anti-CCR10 CAR-T cells with or without knockout of CCR10, empty CAR, and un-transduced T-cells were incubated with MM.1S-luciferase cells for 24 h. Tumor lysis was measured by luminescence (*n* = 3 technical replicates). Error bars represent +/− SD. Source data are provided as a Source data file. **G** Average LFQ intensity across cell lines of proteins >2 SD above the mean versus average unique peptides identified in each line. Source data are provided as a Source data file. **H** Bioinformatic strategy to nominate possible high-abundance locking-on antigens. **I** Absolute quantification by flow cytometry for CD38 and CD48 antigen density across 3 myeloma cell lines (MM.1S, OPM-2, AMO1) and CD138+/CD19− myeloma tumor cells from 5 primary patient bone marrow specimens. Datapoints represent averages of independent replicates for cell lines or technical replicates for primary myeloma samples. *p*-value by two-sided *t*-test. Source data are provided as a Source data file. For boxplots in **C** and **I**, upper and lower hinges correspond to 25 and 75 percentiles, upper and lower whiskers extend to highest and lowest values within 1.5* IQR of the hinge, and center line corresponds to the median.

We next turned our attention to possible immunotherapeutic strategies revealed by our surface profiling. We first integrated our proteomic data with publicly available transcriptome datasets to create a ranking system for possible single-antigen immunotherapy targets, based on five criteria related to surface abundance and specificity for plasma cells (Fig. 2A; Supplementary Table 1). Emphasizing the validity of this strategy, four of the top six targets by our ranking either already have FDA-approved therapeutics or are being clinically investigated in myeloma: BCMA (*TNRFSF17* gene), TACI (*TNFRSF13B*), Integrin beta-7 (*ITGB7*), and CS-1/SLAMF7 (*SLAMF7*) (Fig. 2A). CD38, CD138/SDC1, LY9/CD229, CD48, and GPRC5D also ranked highly (Supplementary Data 2). Based on these results, we probed other high-scoring proteins found in our proteomic data that, to our knowledge, have not yet been explored as therapeutic targets in myeloma. We were most intrigued by CCR10, a chemokine receptor previously shown to be expressed on plasma cells and thought to relate to homing to resident tissues^17^. We found this gene to be robustly expressed on myeloma plasma cells per the CCLE but with minimal expression on other tumor cell lines (Supplementary Fig. 2A). Data from GTEx also suggest low mRNA expression on non-hematopoietic tissues, while data included in the Human Blood Atlas suggest markedly higher mRNA expression on plasmablasts than other hematopoietic cells, with the exception of some T-cell subtypes, including T regulatory cells, consistent with prior literature^18^ (Supplementary Fig. 2A). We verified markedly increased CCR10 expression on myeloma cell lines compared to B-cell malignancy lines (Supplementary Fig. 2B), and, importantly, we confirmed expression of CCR10 on CD138+ plasma cells from all ten patient bone marrow aspirates profiled (Fig. 2B, Supplementary Fig. 2C, D). Simultaneously, we confirmed no detectable expression of CCR10 on other peripheral blood hematopoietic cells (Supplementary Fig 2E). Finally, we found that high tumor *CCR10* expression is predictive of worse overall survival in myeloma patients and *CCR10* expression is increased in relapsed myeloma tumors relative to newly diagnosed, as well as high-risk genotypes (Fig. 2C, D, Supplementary Fig. 3A–G). Together, these findings suggest CCR10 as a promising immunotherapy target in myeloma.

As cellular therapies are the most promising new frontier in myeloma therapy^19^, we next sought to develop a proof-of-principle chimeric antigen receptor (CAR) T-cell versus CCR10. We took advantage of known CCR10 biology, where CCR10 serves as the sole receptor for the chemokine CCL27 (ref. ^20^). Analogous to natural ligand designs against other targets^21^, we fused full-length CCL27 sequence to a 4-1BB-based, 2nd generation CAR and lentivirally transduced primary human CD8+ T cells (Fig. 2E). CAR transduction in Jurkat cells confirmed strong activation after exposure to MM.1S myeloma cells; however, we noted baseline activation above that of control anti-BCMA CAR-transduced Jurkats (Supplementary Fig. 4A). In addition, when transduced into primary T-cells, we initially had difficulty in manufacturing, suggesting that CCR10 upregulation on T-cells during CD3+/CD28+ stimulation (Supplementary Fig. 4B) was leading to T-cell “fratricide”. As with other targets with similar phenotypes^22^, we were able to overcome this hurdle by Cas9 ribonucleoprotein-based knockout of *CCR10* before lentiviral transduction (Supplementary Fig. 4C). Encouragingly, we found that CCL27-based CAR-Ts with *CCR10* knockout exhibited in vitro killing activity against MM.1S myeloma cells (Fig. 2F, Supplementary Fig. 4D, E). These data serve as a proof of concept for further investigation of CCL27-CAR-T-cells as an immunotherapeutic in myeloma.

We also evaluated TXNDC11, a poorly characterized protein with sparse literature revolving around its role in endoplasmic reticulum (ER) associated protein degradation^23,24^. Notably, proteins with known ER localization may also have surface components; for example, the ER-resident HSP70 isoform BiP/GRP78 can be found at the cell surface in myeloma and serve as an immunotherapy target^25^. Furthermore, immunofluorescence data in the Human Protein Atlas^26^, as well as bioinformatic prediction from COMPARTMENTS^27^, both localize TXNDC11 at least partially to the plasma membrane. In addition, per the Cancer Dependency Map^28^, myeloma plasma cells appear genetically dependent on TXNDC11 for proliferation (Supplementary Fig. 5B). As no antibody reagents exist that are suitable for TXNDC11 flow cytometry, we performed confocal microscopy on AMO1 and MM.1S to evaluate TXNDC11 localization. As expected, the majority of TXNDC11 was localized intracellularly. However, we did observe apparent co-localization of some TXNDC11 with plasma membrane-localized CD38 (Supplementary Fig. 5C). Finally, we also briefly evaluated LILRB4, a known immunotherapy target in acute myeloid leukemia^29^, confirming high expression in myeloma cell lines (Supplementary Fig. 5D). While we highlight these three proteins, additional high-scoring targets from our integrative analysis may also warrant further follow-up in myeloma therapy.

### Characterizing the most abundant myeloma surface proteins for biological signatures and potential co-targeting

To identify biological roles of the most highly abundant surface proteins on myeloma cells, we evaluated surface proteins >2 SD above the LFQ mean (Fig. 2G, Supplementary Fig. 6A). 4F2 (encoded by *SLC3A2*) and LAT1 (*SLC7A5*), which together comprise the heterodimeric large neutral amino acid transporter CD98, as well as the neutral amino acid transporter AAAT (*SLC1A5*), appear to be the most abundant proteins on the myeloma cell surface. This observation is potentially consistent with the critical role of protein synthesis in plasma cell biology. Other high-abundance plasma membrane proteins govern homeostatic mechanisms common across most human cells (AT1A1, AT1B3, TFR1/CD71). However, several of the other most highly abundant surface proteins carry specific functions in cellular signaling, including CD138/SDC1, CD47, CD38, ICAM1/CD54, ITA4/CD49d, and CD48/SLAMF2.

We also reasoned that identifying the most highly-expressed proteins on the surface of plasma cells may be advantageous for certain therapeutic designs. We illustrate one potential approach in Supplementary Fig. 6B. In this strategy, a high-abundance, but relatively non-specific, surface antigen could be used to increase avidity of CAR-T cells targeting a highly specific myeloma antigen such as BCMA. By increasing T-cell dwell time on tumor, this approach could potentially enable CAR-T killing at lower antigen densities, which recent studies have suggested may be an important determinant of efficacy^30,31^. We interrogated our data to identify proteins with (1) high abundance on plasma cells in our data, (2) confidently localized to cell surface, (3) high mRNA expression in myeloma patient tumors; (4) high mRNA expression on myeloma cell lines (see “Methods”). This analysis identified 14 potential locking-on protein candidates (Fig. 2H).

Integrating our analyses, we specifically focused on two targets for further evaluation: CD38 and CD48. We chose these proteins as others were expressed on at least some non-hematopoeitic tissues per GTEx (Supplementary Fig. 6C). While CD38 is a well-known monoclonal antibody (mAb) target in myeloma^32^, and CD48 has been previously investigated as an antibody-drug conjugate target^33^, for both markers there are toxicity concerns as standalone CAR-T targets given widespread expression on other hematopoietic cells (Supplementary Fig. 6D–F)^13,33^. To evaluate which of these proteins is more highly expressed at the myeloma cell surface, we used calibrated flow cytometry to measure absolute antigen density of both CD38 and CD48 expression. We evaluated myeloma cell lines (MM.1S, OPM-2, AMO1) and primary bone marrow aspirate samples from 5 relapsed/refractory myeloma patients, selected on CD19−/CD138+ plasma cells. Both antigens were expressed at high copy number, consistent with our proteomic findings, but CD48 showed the higher density (range: 59,307–2,769,932 copies/cell vs. 16,251–613,422 for CD38) (*p* = 0.05) (Fig. 2I, Supplementary Fig. 6G). We thus conclude that CD48 may be a particularly strong candidate for an avidity-based strategy to enhance CAR-T activity.

### The myeloma surface proteome is remodeled in the context of proteasome inhibitor resistance

**Fig. 3:**
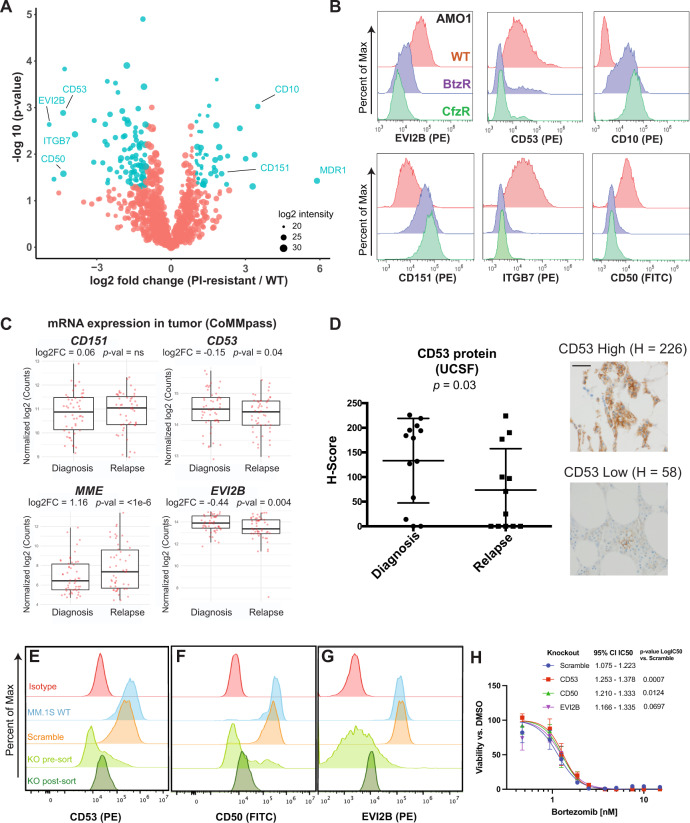
Defining a myeloma surface signature of proteasome inhibitor resistance. **A** Cell surface proteomics was performed on evolved bortezomib-resistant (BtzR) and carfilzomib resistant (CfzR) myeloma cell lines (AMO1 BtzR (*n* = 3); AMO1 CfzR (*n* = 3), L363 BtzR (*n* = 2), L363 CfzR (*n* = 3), RPMI-8226 BtzR (*n* = 1)) and aggregated in comparison to wild-type cell lines (AMO1 (*n* = 3), L363 (*n* = 2), RPMI-8226 (*n* = 1)), *n* denotes number of biological replicates. Significantly changed proteins in PI-resistant lines shown in blue (log_2_-fold change > |1|; *p* < 0.05). Source data in Supplementary Data 3. **B** Validation by flow cytometry of most-changed surface proteins in AMO1 cells. Representative data of *n* = 2 independent experiments. **C** mRNA data in the MMRF CoMMpass database (Release IA14) from paired diagnosis and first-relapse tumor cells (*n* = 50), where all patients had received a PI as part of their induction regimen. *p*-value by two-sided *t*-test. Upper and lower hinges correspond to 25 and 75 percentiles, upper and lower whiskers extend to highest values within 1.5*IQR of the hinge, and center line indicates the median. **D** Immunohistochemistry for CD53 on myeloma plasma cells in bone marrow core biopsies from UCSF patients before and after Btz treatment (*n* = 13 patients). H-scoring (see “Methods”) averaged from two independent hematopathologists (E.R. and S.P.). Magnification = ×60, scale bar length = 100 µm. Error bars represent +/− SD and center line represents the mean. *p*-value by two-sided *t*-test. **E**–**G** Flow cytometry illustrating knockout of CD53 (**E**), CD50 (**F**), and EVI2B (**G**) in MM.1S cells. Representative of *n* = 2 technical replicates. **H** MM.1S engineered with knockouts and scramble guide RNA control were treated with Bortezomib for 48 h (*n* = 3 technical replicates). 95% confidence interval of IC50s from Graphpad (see “Methods”). Error bars represent +/− SD. *p*-values by Extra sum-of-squares F test. For **D** and **H**, source data are provided as a Source data file.

We next proposed that cataloging alterations in the context of proteasome inhibitor (PI) resistance may be relevant for diagnosing this condition, identifying biological strategies to overcome resistance, or developing immunotherapies that selectively eliminate resistant disease. To this end, we performed cell surface proteomic profiling of AMO1, L363, and RPMI-8226 myeloma cell lines previously described to be in vitro-evolved for resistance to either carfilzomib (CfzR) or bortezomib (BtzR)^34^ (Fig. 3A, Supplementary Fig. 7A, B, Supplementary Data 3). Serving as a positive control, the drug efflux pump MDR1 (*ABCB1)* was by far the most increased surface protein in CfzR cells, consistent with our prior results from whole-cell shotgun proteomics^35,36^ (Supplementary Fig. 7C). Aggregating BtzR vs. wild-type data, though, we saw no change in MDR1 (Supplementary Fig. 7D), consistent with prior findings^35^. Instead, across both CfzR and BtzR comparisons we found a signature whereby CD50, CD361/EVI2B, CD53, and Integrin-b7 (ITGB7) were commonly decreased while CD10 and CD151 were increased versus parental. Compared to RNA-seq (Supplementary Data 4), several genes showed evidence of possible post-transcriptional regulation; CD151, for example, showed >2.5 log_2_ fold increased surface protein but essentially no transcript change in AMO1-BtzR cells (Supplementary Fig. 7E). We did not identify significant upregulation of any current myeloma immunotherapy target proteins (Supplementary Data 3). Furthermore, with the possible exception of CD10, we did not identify signatures suggestive of de-differentiation to a more B-cell-like surface protein profile^37^.

Flow cytometry largely validated the extensive surface remodeling uncovered by our proteomic dataset, confirming prominent alterations in CD53, EVI2B, CD50, CD10, and CD151 in both AMO1 (Fig. 3B) and RPMI-8226 (Supplementary Fig. 7F) PI-resistant cells. Our previous analysis indicated that CD53, EVI2B, and CD10 cell surface levels were transcriptionally regulated (Supplementary Fig. 7E). Therefore, to investigate relevance to myeloma patients, we interrogated mRNA expression in paired pre- and post-first relapse tumor cells in the MMRF CoMMpass dataset (release IA14). 94% of these CoMMpass patients were treated with PI as part of their induction regimen. Consistent with our proteomic findings, in relapsed myeloma patient tumor cells we found significant transcript decreases of *CD53* and *EVI2B*, while *MME* (CD10) showed a significant increase (Fig. 3C). *CD151* showed a non-significant increase, though in the context of post-transcriptional regulation protein-level increase could potentially be higher. We further developed an immunohistochemistry (IHC) assay for CD53, confirming decrease on plasma cells in bone marrow core biopsies (*n* = 13) in paired diagnosis and relapse specimens after a PI-containing regimen (Fig. 3D).

While these markers are altered in patient tumors after chronic Btz exposure, expression of these genes at diagnosis did not strongly predict patient outcomes in CoMMpass (Supplementary Fig. 7G). To test the functional implication of identified proteins, we generated CRISPR-Cas9 mediated knockouts of *CD50*, *CD53*, and *EVI2B* in Btz-sensitive MM.1S and tested their effects on drug sensitivity (Fig. 3E-G). Interestingly, all three knockouts led to slightly increased resistance to Btz, with *CD50* and *CD53* knockouts exhibiting modest but significant shifts in IC_50_ (Fig. 3H). However, CD151 overexpression did not alter Btz sensitivity (Supplementary Fig. 7H, I). Together, these results suggest that these surface markers may play a limited role in directly causing PI resistance but are likely best-considered as indirect biomarker candidates of this phenotype.

### Lenalidomide evolved resistance leads to increased CD33 and CD45/PTPRC on myeloma cells

**Fig. 4:**
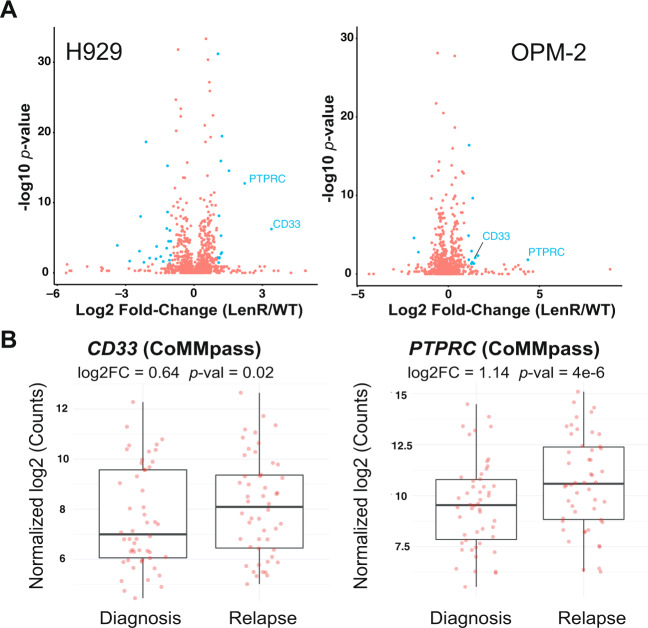
Surface proteomic signatures of lenalidomide resistance. **A** In vitro-evolved lenalidomide-resistant H929 and OPM-2 lines were analyzed by cell-surface proteomics with comparison to parental lines by SILAC quantification (*n* = 4 biological replicates; heavy and light channels swapped for two replicates each). Significantly-changed proteins in blue (log_2_-fold change > |1|; *p* < 0.05 by *t*-test), with only CD33 and PTPRC/CD45 showing common changes between the two lines. Source data in Supplementary Data 6. **B** MMRF CoMMpass patient transcript data confirms significant increase in *CD33* and *PTPRC* at first relapse versus diagnosis (Release IA14, *n* = 50 patients), suggesting that increases in these surface proteins is driven by IMiD resistance. For **B**, upper and lower hinges correspond to 25 and 75 percentiles, center line indicates the median, and upper and lower whiskers extend to highest and lowest values within 1.5* IQR of the hinge.

We further probed surface proteomic changes in the context of evolved resistance to lenalidomide (Len), a thalidomide analog used in both the standard front-line regimen for myeloma patients and maintenance monotherapy. To our knowledge, surface changes resulting from Len resistance have not been previously characterized. We performed CSC proteomics on OPM2 and H929 cell lines in vitro-evolved to become resistant to lenalidomide (LenR) relative to their WT counterparts^38^. While we found broad surface proteomic changes in both cell lines, there was relatively little overlap between the two (Fig. 4A, Supplementary Data 6). The most notable common signature was increased CD33 and CD45/PTPRC in both LenR cell lines (Fig. 4A). Examining CoMMpass data, we confirmed both *CD33* and *PTPRC* transcripts to be significantly increased at first relapse vs. diagnosis in patient tumor cells (Fig. 4B). Plasma cell expression of either of these markers has already been proposed as a poor prognostic factor in newly-diagnosed myeloma^39,40^, potentially consistent with more aggressive disease biology after Len resistance. In terms of therapeutic targeting, while CD45 is expressed at high levels on essentially all non-plasma cell leukocytes, CD33 is a well-known surface target enriched in myeloid malignancies^41^. However, by CSC profiling, CD33 shows low mass spectrometric intensity on myeloma plasma cells (Supplementary Data 1), suggestive of low surface protein expression. Furthermore, per Human Blood Atlas data *CD33* mRNA expression on B-lineage cells is expected to be much lower than that on myeloid cells (Supplementary Fig. 7J). On-target, off-tumor toxicity on non-malignant cells expressing CD33 is already a considerable concern in treatment of myeloid leukemias^42^. Therefore, exploiting CD33 upregulation on lenalidomide-resistant myeloma may prove challenging.

### Short-term drug treatment leads to divergent surface profiles from evolved resistance

Another potential strategy to take advantage of the myeloma surfaceome is to consider co-treatment approaches of small molecules and immunotherapies. For example, our group^43^ and others^44–46^ have used small molecules to increase expression of CD38 on myeloma plasma cells to enhance efficacy of daratumumab. Other examples include small molecule treatment to boost surface BCMA in myeloma^47,48^ or CD22 in B-ALL^49^. Furthermore, it has been suggested that acute responses to PI treatment, in particular marked chaperone upregulation and ER stress response, may translate into mechanisms of long-term cellular adaptation and resistance^50–52^. We therefore examined the effects of short-term (48 h) Btz and Len treatment on the myeloma surfaceome (Supplementary Data 5).

**Fig. 5:**
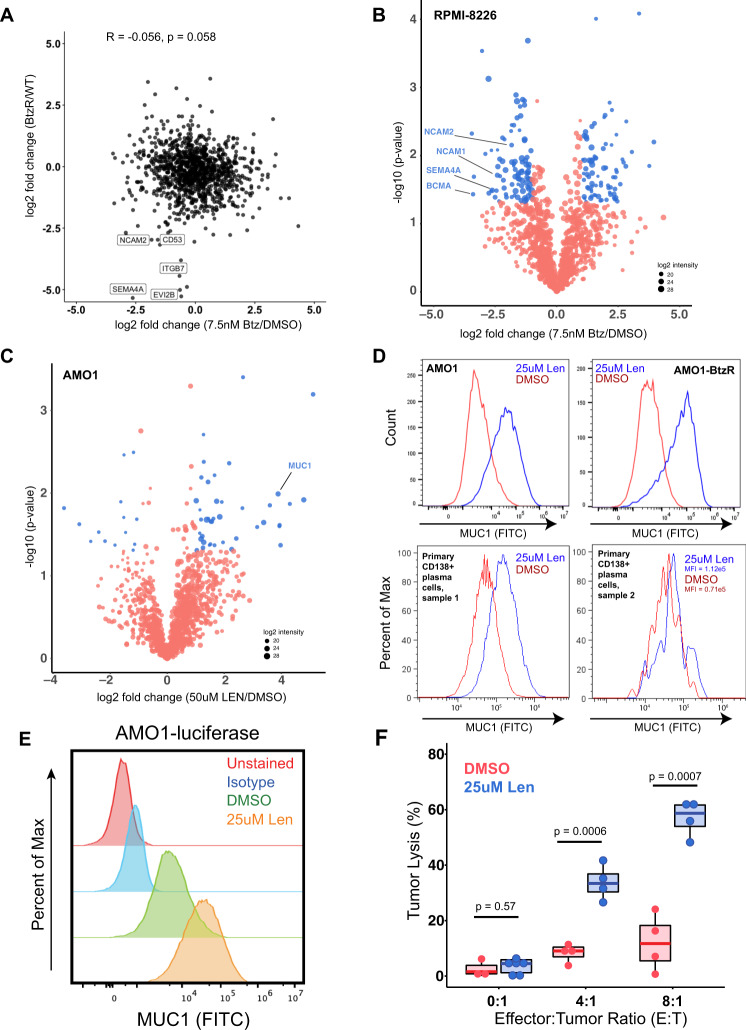
Characterizing myeloma surface proteomic changes in response to acute drug treatment. **A** Correlation in surface proteomic profile between acute Btz treatment in RPMI-8226 cells (7.5 nM, 48 h, *n* = 3 biological replicates) vs. DMSO (*n* = 4 biological replicates) and aggregate BtzR cell line data (as in Fig. 3A) vs. parental. Common changes are observed in some downregulated surface proteins but no significant overall correlation is observed. Pearson correlation and associated *p*-value of significance shown. Source data in Supplementary Data 3 and 5. **B** Volcano plot of RPMI-8226 cells treated for 48 h with 7.5 nM bortezomib, highlighting significantly changed proteins (log_2_-fold change > |1|; *p* < 0.05 in blue). *n* = 3 biological replicates. **C** Similar plot as in **B**, for 48 h treatment with 50 μΜ Lenalidomide in AMO1 cells. *n* = 3 biological replicates. For **B**, **C**, source data in Supplementary Data 5. **D** Validation of increase in surface MUC1 in plasma cells in response to 25 μM Lenalidomide treatment, in both AMO1, AMO1 BtzR, and CD138+ myeloma cells in two patient bone marrow aspirates. All plots representative of *n* = 3 (cell line) or *n* = 2 (primary sample) technical replicates. **E** MUC1 expression on cells treated with DMSO or Lenalidomide prior to incubation with anti-MUC1 CAR-T cells (representative of *n* = 2 independent experiments). **F** Percent tumor lysis of AMO1-luciferase cells after incubation with Anti-MUC1 CAR-T cells after 72 h, as measured by luminescence. Anti-MUC1 CAR-T cells more efficiently kill cells pre-treated with lenalidomide, while lenalidomide alone has no cytotoxicity (*n* = 4 technical replicates for E:T = 4:1 and E:T = 8:1, *n* = 8 technical replicates for E:T = 0:1, *p*-values by Student’s *t*-test). Source data are provided as a Source data file. Upper and lower hinges correspond to 25 and 75 percentiles, upper and lower whiskers extend to highest and lowest values, and center line indicates the median.

We first noted that surface protein changes after acute Btz treatment at 7.5 nM for 48 h in RPMI-8226 cells, when compared to BtzR vs. WT data aggregated across cell lines, did not show significant correlation (*R* = −0.056; *p* = 0.058) (Fig. 5A). The most apparent commonalities were in downregulated proteins, including CD53, NCAM2, and SEMA4A. While we did not observe an increase in any current immunotherapy targets after acute Btz treatment, we surprisingly noted a decrease in surface BCMA in RPMI-8226 cells (Fig. 5B). We further confirmed this finding in another cell line, MM.1S, by flow cytometry (Supplementary Fig. 8A, B).

We similarly investigated short-term treatment with Len. After 48 h of 50 μM treatment in AMO1 we again found broad alterations to the surfaceome (Fig. 5C). Interestingly, we did not observe any alteration in CD33 or PTPRC/CD45. Among the most upregulated proteins was Mucin-1 (MUC1), a known therapeutic target in multiple myeloma^53^. By flow cytometry we confirmed MUC1 surface changes after short-term Len in both AMO1 as well as primary CD138+ plasma cells from two patients (Fig. 5D). Exploring this result, we further found that Len significantly increased killing of AMO1-luciferase cells by previously developed anti-MUC1 CAR-Ts^54^, under conditions where Len alone did not lead to any cell death (Fig. 5E, F, Supplementary Fig. 8C). Co-treatment with this first-line small molecule may thus be considered for future investigation in combination with MUC-1 directed therapies.

Intriguingly, myeloma surface markers show wide variability in expression changes when aggregated across treatment with several different small molecules (Supplementary Fig. 8D–H). For example, TXNDC11 and CCR10 protein levels appear stable across drug treatments in comparison to canonical myeloma markers BCMA and CD138/SDC1. Together, these findings confirm that acute drug treatment and long-term resistance lead to rare commonalities but, in general, largely divergent effects on the myeloma surfaceome.

### Micro-method for small sample input and primary myeloma surface profiling

**Fig. 6:**
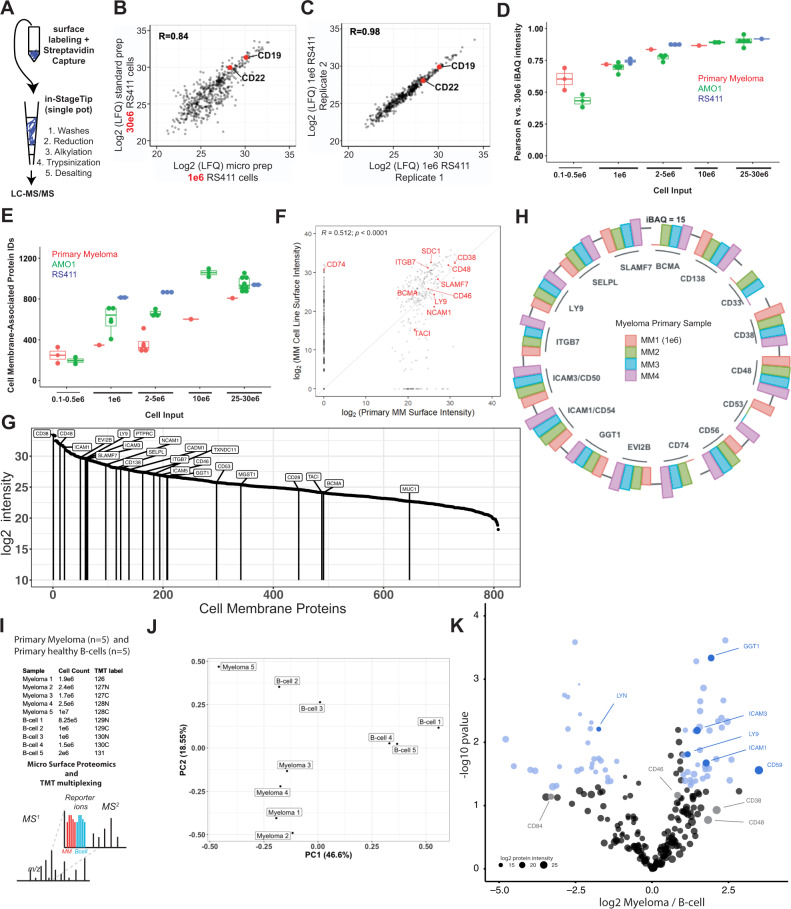
Micro-protocol for cell surface proteomics. **A** Schematic of micro sample preparation method using an InStageTip approach for all steps after surface glycoprotein biotinylation on live cells. **B** Quantitative comparison of LFQ intensity for identified proteins using 30e6 cells in the standard, macro-protocol, versus 1e6 cells using our micro-protocol. LFQ values averaged from *n* = 2 biological replicates per preparation method; performed with RS4;11 B-ALL cells. **C** The micro method demonstrates excellent reproducibility across both biological replicates at 1e6 cell input. **D** Pearson *R* values of indicated cell inputs of RS411, AMO1, or primary myeloma against the 25e6–30e6 proteomic sample from the same cell line or primary sample. Data points for AMO1 and RS411 represent biological replicates compared to one of the 25e6–30e6 cellular input biological replicates. For primary myeloma, one primary sample MM1 was titrated at inputs of 25e6, 10e6, 5e6, 1e6, 0.5e6, and 0.1e6. **E** Total number of cell membrane-associated proteins identified using the micro-method at various cell inputs, on AMO1, RS411, and CD138+ tumor cells isolated from four relapsed/refractory myeloma patients. Samples underlying datapoints in E are from the same samples used for correlation analysis in D, with the addition of the 25e6–30e6 biological replicate for AMO1, RS411, and MM1 used for correlation as well as three additional primary myeloma patient samples (MM2, MM3, MM4) which were collected with total cellular inputs between 2e6–5e6. For **B**–**E**, source data are provided as a Source data file. **F** Quantitative comparison of identified cell membrane proteins in the 25e6 cell input primary sample (*x*-axis) versus averaged over the four profiled myeloma cell lines (*y*-axis). Pearson *R* reported. Source data available in Supplementary Data 1 and 7. **G** Cell membrane protein intensities in the MM1 sample with 25e6 input. Relevant therapeutic targets and other antigens noted in the manuscript are specifically labeled. **H** Immuno-targets and biomarker candidates identified using micro scale proteomics on the surface of CD138+ myeloma cells isolated from four patient samples. MaxQuant iBAQ absolute quantification intensity reported. For **G**, **H**, source data in Supplementary Data 7. **I** Schematic of primary myeloma vs. B-cell TMT proteomics experiment. Micro protocol was performed CD138+ cells isolated from an additional five primary myeloma patient samples and B-cells isolated from five healthy donors. Peptides were labeled with TMT-10plex reagents and combined prior to fractionation and LC-MS/MS. **J** PCA of myeloma and B-cell primary proteomic samples from TMT multiplex. **K** Comparison of Myeloma and B-cell membrane associated proteomes validates GGT1, ICAM3/CD50, ICAM1/CD54, and LY9 as some of most upregulated primary myeloma surface proteins relative to B-cells (log_2_-fold change > |1 | ; *p* < 0.05 in blue). For **J**, **K**, source data in Supplementary Data 10. For boxplots in **D**, **E**, upper and lower hinges correspond to 25 and 75 percentiles, upper and lower whiskers extend to highest and lowest values within 1.5* IQR of the hinge, small-sized points indicate means, and center line corresponds to the median.

Myeloma bone marrow aspirate specimens available for research or diagnostic purposes typically yield in the range of 0.5e6–5e6 CD138+ malignant plasma cells. We thus sought to develop a streamlined, miniaturized CSC method amenable to reduced sample inputs, thereby allowing for more routine application to primary samples. We reasoned that adapting the InStageTip strategy^55^ could meet this need. This one-pot approach, which minimizes sample losses by eliminating many steps of handling and vessel transfer, was previously shown to allow for large decreases in shotgun proteomic sample input on whole-cell lysates. In our adaptation of this method for cell surface proteomics, following surface biotinylation of live cells, streptavidin bead capture, and bead washes, all steps are performed in a single P200 tip (Fig. 6A). We first applied this micro-method to RS4;11 B-ALL cells. With the micro method and 1e6 cell input, we were able to identify 830 and 799 membrane-associated proteins in each replicate, compared to 936 and 942, respectively, obtained with the standard macro method on 30e6 cell input. We further saw consistent quantification between the micro and macro method (Pearson *R* = 0.84) and excellent quantitative reproducibility between biological replicates of the micro method on 1e6 cells (*R* = 0.98) (Fig. 6B, C).

Next, we applied the micro method to range of cellular inputs of AMO1 cells. At 1e6 input, we were able identify an average of 601 membrane-associated proteins (Fig. 6D, E, Supplementary Data 9), with good reproducibility against the macro prepped 30e6 sample (average *R* = 0.693) and between 1e6 and 3e6 inputs (*R* = 0.85) (Fig. 6D, Supplementary Fig. 9A, B). We therefore attempted to apply our micro-CSC method to primary myeloma plasma cells, using cells obtained from four patient bone marrow aspirates via CD138+ magnetic bead isolation. In three cases we isolated a typical yield of ~3e6–5e6 plasma cells; however, in sample MM1, with a very rare malignant pleural effusion, we were able to obtain 50e6 cells. This allowed us to perform a titration experiment, testing the micro method on primary myeloma cells across a range of inputs, 25e6, 10e6, 5e6, 1e6, 0.5e6, and 0.1e6 cells, and compare the number of primary IDs and correlations. Primary myeloma samples suffer a reduction in the number of IDs relative to cell lines, however, the quantification against the benchmark 25-30e6 cell input sample achieves good reproducibility at the 1e6 input level (Fig. 6D, E, Supplementary Fig. 9C). The micro one-pot approach on 25e6 cellular input quantified 808 total membrane-annotated proteins (Supplementary Fig. 9C, Supplementary Data 7), and showed moderate positive correlation (*R* = 0.51; *p* < 1e4) between surface protein quantification in myeloma cell lines and in this primary sample, with relatively few surface proteins found on the primary sample not identified in myeloma cell lines (Fig. 6F). Encouragingly, we were able to identify many known myeloma immunotherapy targets in each of the four primary samples (Fig. 6G, H, Supplementary Fig. 9D–F). Notably, we identified peptides from LILRB4, GGT1, and CCR10, further validating expression of surface markers on primary myeloma cells that we initially highlighted from cell line analysis (Supplementary Fig. 9G–I). As a final test of our approach, we performed Tandem Mass Tag (TMT) isobaric labeling and mass spectrometry for five additional primary myeloma samples and five primary B-cell samples isolated from healthy donors, with input ranges of 0.8–2.5e6 for all samples except one myeloma sample, which yielded 10e6 cells (Fig. 6I, J). GGT1, ICAM3/CD50, and ICAM1/CD54, earlier identified in the myeloma cell line proteomics (Supplementary Data 1), were among the most enriched proteins on the primary myeloma cell surface relative to B-cells (Fig. 6K). Taken together, these results illustrate the potential of a miniaturized cell surface capture method to enable more routine surface proteomic profiling of primary myeloma specimens.

## Discussion

Here we used a proteomic approach to delineate the cell surface landscape of multiple myeloma plasma cells. In these myeloma models, our results characterize the distinct myeloma cell surface repertoire as well as define surfaceome remodeling in the context of resistance to and acute treatment with commonly used small molecule therapeutics. Our findings suggest additional immunotherapy targets in this disease, reveal possible protein markers of resistance, and provide a unique resource for probing plasma cell biology given the numerous physiologic processes governed by cell surface proteins. Furthermore, we modify a common cell surface proteomic method which, with further refinement, may more readily expand surfaceome profiling to primary tumor samples.

Notably, our dataset is valuable as it extends to far more proteins simultaneously than are accessible by flow cytometry or CyTOF. On the other hand, for surface proteins present at low copy number, or with no glycosylation, sensitivity of detection by CSC proteomics is certainly lower than these other approaches. Furthermore, we cannot exclude the possibility of false-positive hits due to background intracellular protein labeling. Therefore, this broad dataset ultimately serves as an important complement to targeted antibody-based approaches.

We believe that particular utility of this resource comes from its ability to identify surface proteomic signatures that could not be predicted from RNA-seq alone. For example, our identification of surface proteins most highly expressed on plasma cells (Fig. 2C–E) cannot be directly extrapolated from transcriptome data. Furthermore, we find evidence of potential post-transcriptional regulation of many surface proteins in the drug-resistant setting (Supplementary Fig. 6E).

In using a previously generated leukemia surface proteomic dataset to compare against the myeloma surfaceome generated here (Fig. 1E), we note that it is impossible to completely remove batch effects. However, we observed that ARH-77 data, obtained concurrently with myeloma cell lines, clustered closely with donor B-lymphoblast data, obtained two years earlier with B-ALL lines. Furthermore, batch effect quantification found that “tissue” was the greatest driver of variance among the dataset (Supplementary Fig. 10A, B).

We further integrate our proteomic data with RNA-based datasets to assist in the identification of immunotherapy targets in myeloma (Fig. 2A). Our proof-of-principle CCL27-CAR-T cells versus CCR10 show initial evidence for specificity of cytotoxicity (Fig. 2F). However, we acknowledge that in vitro potency is relatively limited, with cytotoxicity achieved at E:T ratios of 1:1 or higher (Fig. 2F), suggesting that additional CAR engineering is required to generate a candidate for in-depth preclinical development. Furthermore, a limitation of CCR10 as a target include that it is expressed on activated T-cells. However, other successful CAR-Ts have been built versus similar targets (e.g., CD70 (ref. ^56^), ITGB7 (ref. ^57^), or LY9 (ref. ^58^), and it is now well-demonstrated that CRISPR-based knockouts can be incorporated into CAR-T manufacturing approaches^59^. Another possible limitation is that CCR10 has low-level expression on some other immune cells beyond plasma cells (Supplementary Fig. 2A). However, we note that CCR10’s off-tumor expression profile is comparable to other clinically-investigated myeloma CAR-T targets SLAMF7 and CD138 (Supplementary Fig. 11A–C), and therefore is not anticipated to be particularly unfavorable. Yet we cannot rule out that these features will lead to complications in manufacturing or additional toxicities. Other antigens emerging from our scoring system may also have promise and stand as a resource for others in the community. In parallel, modulating CAR binding avidity has recently been proposed as a method to precisely tune CAR-T activation^60^. Our proposal to use CD48/SLAMF2 binding to increase avidity, and thereby extend functional activity, of BCMA CAR-Ts requires future experimental exploration.

In terms of other study limitations, due to the sample input required for cell surface proteomics, the majority of our initial surface profiling is performed in myeloma cell lines. While we know these models are only partially representative of true patient tumors^61^, our identification of nearly all canonical myeloma surface markers and immunotherapy targets on cell lines (Fig. 1C; Supplementary Data 1) increases confidence in the broader applicability of our findings. In addition, many of the drug-resistance or drug-treatment signatures we identified were consistent with those observed in primary patient tumors, either at the transcript (MMRF CoMMpass) or protein (our studies here) level. While our genetic modulation of surface-protein encoding genes revealed only modest direct impacts on PI sensitivity (Fig. 3H), more broadly these antigens are known to be important for cell-cell interactions within the immune microenvironment^62^. Future investigation will employ immunocompetent mouse models to investigate impacts on disease progression and response to therapy.

In general, broadly extending surface proteomic profiling to primary plasma cells would certainly be a boon for myeloma research. Our miniaturization of the CSC method provides a step toward allowing more routine profiling of primary samples, but further optimization is clearly needed. Recently, a method utilizing automated liquid handlers was described, obtaining high-quality data from only 1e6 murine B-cells^63^. However, this approach is not necessarily optimal as it requires specialized equipment only available in a handful of labs. While we acknowledge a limitation of our study is that the micro-method may not always lead to a large increase in number of surface protein identifications versus the macro-approach, we still believe that the micro-method will carry advantages for other groups performing surface proteomics given a simplified, streamlined protocol and no apparent disadvantages. We also note that with low primary sample inputs quantitative precision is decreased (Fig. 6D, E), suggesting this approach may be most useful for discovering expression of novel targets. Despite these limitations, we show that our micro-method can be used to proteomically profile the cell surface of low-to-moderate input primary plasma cell specimens, laying the groundwork for more routine surfaceomics profiling.

In conclusion, we provide a unique resource for the myeloma research community through our surfaceome profiling approaches. Through further method advancement, our goal is to ultimately bring this strategy to more widespread use in basic, translational, and clinical studies in myeloma.

## Methods

### Human research participants

All human samples were obtained in accordance with the Declaration of Helsinki and through protocols approved by the UCSF Committee on Human Research Institutional Review Board (IRB). These include all primary myeloma bone marrow aspirate specimens obtained through the UCSF Multiple Myeloma Translational Initiative (MMTI) protocol (UCSF IRB approval 10-00545) and bone marrow core biopsies obtained through the UCSF General Hematopathology Protocol (Hemepath) (UCSF IRB approval 10-01080) (Supplementary Table 2).

Informed consent was obtained from all myeloma patients contributing bone marrow aspirate or other biofluid samples under the MMTI protocol. Patients consented under the MMTI protocol specifically agree to “information about you and your illness, including your age, sex, race, diagnosis, stage of disease at diagnosis, symptoms at diagnosis, prior treatments, and response to treatments, will be entered into a secure database” for use in research. Patients participating in research were not compensated for participation.

Per IRB approval of the Hemepath protocol, the informed consent requirement has been waived for archived bone marrow core biopsies obtained from patients greater than 6 months prior to analysis for research; only core biopsy samples greater than 6 months in age are analyzed here (Fig. 3D). Informed consent was waived due to the following IRB accepted criteria: “Many subjects are no longer being followed at the institution or are deceased”; “The attempt to contact subjects poses a greater risk than this study”; and “The large number of records required makes it impracticable to contact all potential subjects”. The IRB-approved purpose of this study is to use “Remainder fresh clinical samples (e.g., bone marrow aspirate not needed for clinical purposes to be discarded) and paraffin-embedded tissue will be examined with immunohistochemical stains and/or molecular/cytogenetic techniques (e.g., fluorescent situ hybridization, polymerase chain reaction, other molecular techniques) for both known and emerging molecular markers, biologic characteristics, and genetic/chromosomal abnormalities. Results may be correlated with morphologic, flow cytometric, laboratory, and clinical findings. Data will be compiled to identify biologic characteristics of a disorder and/or markers that may be able to guide diagnostic decisions and may provide prognostic information that may be useful in therapeutic decisions. In addition, biologic markers may be identified that can serve as therapeutic targets”.

Normal donor B-cells and T-cells were obtained from fully de-identified peripheral blood specimens commercially purchased from Vitalant (San Francisco, CA).

### Cell lines

All cell lines were grown in RPMI-1640 media with 10% FBS. PI-resistant cells derived from cell lines AMO1, L363, RPMI-8226, and ARH-77 were grown in 90 nM Bortezomib (Btz) or Carfilzomib (Cfz)^64^. Lenalidomide-resistant cell lines H929 and OPM-2 were grown in increasing concentrations of lenalidomide and removed from drug for 5–7 days before use in cell surface proteomics^38^. Cell lines were verified by DNA short tandem repeat testing and assessed as mycoplasma negative. RS4;11 and MM.1S cells were obtained from ATCC (cat# CRL-1873, CRL-2974). AMO1, KMS12-PE, and RPMI-8226 were obtained from DSMZ (cat# ACC-538, ACC-606, ACC-402).

### Cloning and lentiviral expression of CD151

pLEX-CD151 was cloned using modified pLEX donor vector without uORF (Addgene #120558) and mEmerald-CD151-7 Plasmid (Addgene #54032) using Takara In-Fusion cloning kit (Takara 638943). Cloning priers used were: Forward: 5′-CTCTACTAGAGGATCCATGGGTGAGTTCAACGAGAAGAAGAC-3′ and Reverse: 5′-CTGGACTAGTGGATCTTAGTAGTGCTCCAGCTTGAGACTCC-3′. CD151 or EV (pLEX backbone with no insert) cell lines were produced by lentivirus transduction. Lentivirus production was performed using 293t packaging system with p8.91, pMD2.G, and pCEP4-tat packaging plasmids.

### Cas9 knockouts

Cas9 protein and sgRNA or scramble sgRNA were mixed in 1:1 molar ratio (Synthego Corporation) and incubated at 37 °C for 10–15 min. 1e6 of either primary CD8+ T cells or MM.1S cell line were spun down and washed with PBS, resuspended in 20 μL of P3 nucleofection/solution plus cas9/sgRNA mixture (P3 Primary Cell 4D-Nucleofector^TM^ X Kit S) for primary T cells or SF Cell Line 4D-Nucleofector^TM^ X Kit S (Lonza) for MM.1S cell lines, and nucleofected using EO-115 or DS-137 nucleofection program in Lonza 4D-Nucleofector, respectively. 80 µL of warm (CTS™ OpTmizer™ T Cell Expansion SFM, Gibco™) or RPMI 1640 media (Gibco) was plated into each well and incubated at 37 °C for 15 min, then transferred to a 6-well plate supplemented with IL-7 and IL-15 for T-CD8+ T cells to recover for 48 h. Then, based on the surface expression by flow cytometry, negative clones were sorted using FACSARIA II flow cytometer (BD Biosciences). sgRNA sequences were obtained from Brunello Library^65^ as follows: CCR10: CTGTCGCCTCATCTTCCCCG, TATCAGCGCCGACCGCTACG; CD53: CCGTTACCACTCAGACAATA, EVI2B: CAACTGTCAAAAATTCACCT; CD50 (ICAM3): CGGGGACACGCTAACGGCCA. Negative Control, scrambled sgRNA#1 and #2 mod-sgRNA specified by Synthego Corporation.

### CCL27-CAR-Jurkat activation assay

For all in vitro Jurkat CAR-T cell stimulations, 1e6 Jurkat cells were co-cultured with target MM1.S cells at a 1:1 ratio in a 6-well plate. Cells were analyzed at 24 h for CD69 expression using an APC or PE-conjugated CD69 antibody (BD Biosciences clone FN50) at 1:20 dilution via flow cytometry with a Cytoflex Flow Cytometer (Beckman).

### CAR-T manufacturing

The signaling components for empty and CCL27 CAR, including the CD8 hinge and transmembrane domain, 4-1BB co-stimulatory domain, and CD3ζ signaling domain are identical to those utilized in the clinically approved CD19-directed CAR construct tisangenlecleucel. CCL27 sequence was cloned into the CAR backbone plasmid using Gibson Assembly protocol. Primary human T cells were purified from the leukapheresis products of anonymous healthy blood donors using RosetteSep™ Human CD8+ T Cell Enrichment Cocktail and EasySep kit (STEMCELL Technologies, Inc.) under an institutional review board–exempt protocol in accordance with the U.S. Common Rule (Category 4) and were resuspended in T cell media (CTS™ OpTmizer™ T Cell Expansion SFM, Gibco™) supplemented with 5% of filtered, heat inactivated and sterile human AB serum, 1% Pen-strep, and 1%, glutamax 1% (Gibco™), IL-7 and IL-15 were added to media at 10 ng/mL concentration (PeproTech). For CCL27 CAR, CRISPR-Cas9 KO were performed as described above. Primary T-cells rested for at least 60 min at 37 °C before stimulation with Dynabeads TM Human T-activator CD3/CD28 (Gibco). On day 1, viral supernatant containing second generation of lentivirus enveloped and packaging plasmid (pMD and psPAX2 [addgene Plasmid #12259 and #12260, respectively]) was added to T-cells for 24 h and then washed using PBS. On day 4, beads were removed and transduction efficiency was determined using GFP detection for CCL27 CAR and Fluorescein (FITC) AffiniPure F(ab’)_2_ Fragment Goat Anti-Human IgG, Fcγ fragment specific (Jackson Immunoresearch Laboratories, Inc) for MUC-1 CAR. On day 6, c-myc Antibody, anti-human/mouse/rat, Biotin (Miltenyi Biotec) was used to label myc-Tag to enrich CCL27-CAR or empty CAR, and Biotin-SP (long spacer) AffiniPure Goat Anti-Mouse IgG, F(ab’)_2_ fragment specific for MUC-1 CAR to magnetically enrich CAR positive population using Anti-biotin Microbeads, MS and LS Columns, and a miniMACS or MACS separator (Miltenyi Biotec). CAR-T cells were expanded and CAR expression was verified once again before running cytoxicity assays.

### CAR-T cytotoxicity assay

CCL27-CAR-T cells were mixed with MM.1S-luciferase cells at indicated ratios for 24 h and MUC1-CAR-T cells were mixed with AMO1-luciferase for 72 h. Cells were plated in 200 μL of T-cell media in 96-well plates. Cytotoxicity was measured by bioluminescence, with 150 μg/mL of d-luciferin (Gold Biotechnology) added to media prior to 10 min incubation at room temperature. Luminescence was read with a GloMax Explorer (Promega).

### Myeloma patient sample processing

Fresh de-identified primary myeloma patient BM samples were obtained from the UCSF Hematologic Malignancies Tissue Bank in accordance with the UCSF Committee on Human Research-approved protocols and the Declaration of Helsinki. BM mononuclear cells were isolated by density gradient centrifugation with Histopaque-1077 (Sigma-Aldrich), and washed with 10 mL DPBS 3 times. For small-molecule perturbation experiments, mononuclear cells were plated in IL-6 supplemented medium (RPMI-1640, 10% FBS, 1% penicillin/streptomycin, 2 mM glutamine, with 50 ng/mL recombinant human Il-6 (ProSpec)) at 2e5 cells per well in a 96-well plate and incubated at 37 °C, 5% CO_2_ overnight prior to drug treatment and processing for flow cytometry as described below. For mass spectrometry, isolated mononuclear cells were taken directly from histopaque isolation into CD138 magnetic bead isolation using EasySep Human CD138 Positive Selection Kits as per manufacturer recommendations (StemCell Technologies 17877), followed by surface glycoprotein labeling as described below.

### Primary B-cell Isolation

PBMCs were separated from 15 mL of peripheral blood using Lymphoprep (Stemcell 29283-PIS) following manufacturer recommendations. Briefly, 15 mL of Lymphoprep at room temperature was added to 50 mL Falcon tubes. 15 mL of peripheral blood was mixed with 15 mL of RPMI-1640 media, and then layered on top of Lymphoprep prior to centrifugation at 800 × *g* for 30 min at RT without brake. Opaque layer containing Bone Marrow Mononuclear Cells (BMMCs) was retained and washed with D-PBS, followed by Red Blood Cell Lysis. B-cells were isolated using Easysep Human B-cell Isolation Kit (STEMCELL cat# 17954) following manufacturer recommendations prior to CSC Micro Proteomics and TMT labeling.

### Flow cytometry

Cells were resuspended in FACS buffer (5% FBS in D-PBS) and stained with antibodies for 1–2 h on ice, washed with FACS buffer, and then resuspended in FACS buffer. For experiments where live cells populations were studied, cells were resuspended in FACS buffer. Samples were analyzed using either a Cytoflex Flow Cytometer (Beckman Coulter) or FACSAria II flow cytometer (BD Biosciences). Data analysis was done using FlowJo software, v10.8.1 or v8.8.1. For analyses including live/dead stains, cells were resuspended in FACS buffer containing Sytox Green or Sytox Red (Thermo). For drug treatment experiments, cells were plated into 96-well plates and treated for 48 h with compounds unless otherwise noted. For quantitative flow cytometry, antibodies used were FITC Mouse anti-human CD48 (BD Biosciences, clone TU145), FITC Mouse anti-human CD38 (BD Biosciences, clone HIT2), FITC Mouse IgG1k isotype control (BD Biosciences, clone 27-35), and unstained, respectively. using calibrated beads (Bangs Labortories), 200 µL of FACS buffer + 1 drop of beads per well (one wells for blank beads, and other for FITC-Beads). Compensation was performed using UltraComp eBeads™, compensation Beads (Invitrogen). Calibration curve for quantification beads were performed on the Quantitative software QuickCal v2.3 (Bangs Laboratories, Inc.) before FITC analysis on multiple myeloma cell lines. Additional antibodies used are as follows: CD138 (BD Biosciences, 562097, 552026), BCMA (BD Biosciences, 552026, BioLegend 357504), CD53 (BD Biosciences, 555508), CD10 (BD Biosciences, 561002), CD151 (BD Biosciences, 556057), CD50/ICAM3 (BD Biosciences, 555958), ITGB7 (BD Biosciences, 555945), GITR/CD357 (BioLegend 311604), MUC1/CD227 (BD Biosciences, 559774), CCR10 (BD Biosciences, clone 1B5), CD19 (BD, 555412; Biolegend 363036), CD3 (Biolegend 344840), IgG1 Mouse Control (Biolegend 400141; Biolegend 400196, BD Biosciences 555748), IgG2a Mouse Control (BD 555576), c-Myc, Biotin (Milteny Biotech 130-124-877), Biotin-SP (long spacer) AffiniPure Goat Anti-Mouse IgG, F(ab’)_2_ fragment specific (Jackson Labs, 115-065-072), Fluorescein (FITC) AffiniPure F(ab’)_2_ Fragment Goat Anti-Human IgG (Jackson Labs, 109-096-098). Additional isotype antibodies were ordered from BD Biosciences and BioLegend as per manufacturer recommendations.

### Drug treatments

For cell surface proteomics experiments, 25e6 cells were seeded at 1e6/mL in T75 flasks and treated with compounds for 48 h at the LD_25_ dose. RPMI-8226 cells were treated with 50 μM Lenalidomide (Sigma CDS022536), 7.5 nM Bortezomib (Selleck Chemicals, S1013), or 300 nM CB-5083 (gift of Cleave Biosciences). AMO1 cells were treated with 50 μM Lenalidomide, 750 nM JG-342 (gift of Jason Gestwicki, UCSF), or 250 nM CB-5083. For flow cytometry experiments, cells were plated and treated in 96-well plates with the doses described in figure legends for 48 h prior to flow cytometry analysis unless otherwise indicated. For Bortezomib drug screens, 1000 cells were seeded in 45 μL of media in 384-well plates using the Multidrop Combi (Thermo Scientific). Bortezomib (Selleck Chemicals, S1013) was added 24 h after seeding and viability was measured using Cell-Titer Glo (Promega) 48 h after the addition of drugs using a GlowMax Explorer (Promega).

### Cell surface protein labeling

Cell surface labeling with biotin based on the N-glycoprotein method^11,14^. Cells were harvested and washed twice with cold D-PBS (UCSF CCFAL001). For cell-surface protein labeling, the samples were resuspended in 990 μL cold D-PBS and transferred to a 1.5-mL amber tube. Next, they were oxidized by the addition of 10 μL 160 mM NaIO_4_ (Thermo 1379822) and incubated on a rotisserie at 4 °C for 20 min. Three spin washes were performed each with 1 mL cold D-PBS at 300 × *g* for 5 min to remove the oxidizing reagent. For chemical labeling, cell pellets were resuspended in 1 mL cold D-PBS followed by the addition of 1 μL of aniline (Sigma-Aldrich 242284) and 10 μL biocytin hydrazide (Biotium 90060). Samples were incubated at 4 °C for either 90 min (macro-protocol for sample input 25e6–30e6) or 60 min (micro-protocol for sample input <1e6–5e6) on a rotisserie followed by three more spin washes with cold D-PBS. After the final wash, supernatant was removed, and cell pellet were snap frozen and stored in −80 °C until lysis.

### Macro-cell-surface-capture (CSC)

Samples with cell input of 25e6–30e6 were processed by canonical macro-protocol based on previously published protocols^14^. Labeled cell pellets were lysed in 1 mL buffer containing 2X RIPA (Millipore 20-188), 1X HALT protease inhibitor (Thermo 78430), and 2 mM EDTA. Lysates were sonicated at 1-Hz pulses for 30 s with a probe sonicator and incubated on ice for 10 min. To remove precipitates, samples were spun at 17,200 × *g* for 10 min at 4 °C. To prepare for enrichment of biotinylated proteins, 500 μL of High Capacity NeutrAvidin slurry (Thermo PI29204) were washed three times with 1 mL 2X RIPA/1 mM EDTA buffer in 2-mL chromatography columns (Bio-Rad 7326008) attached to a vacuum manifold (Promega A7231). Clarified lysates were then transferred to the chromatography column and incubated at 4 °C for 120 min on a rotisserie. For the macro protocol, protein-bound beads were transferred to a 10-mL chromatography column (Bio-Rad 7311550) and washed with the following buffers: 50 mL 1x RIPA/1 mM EDTA, 50 mL 1X PBS/1M NaCl, and 50 mL 2 M urea/50 mM ammonium bicarbonate (ABC). After washes, beads were dried fully and transferred to a 1.5-mL tube using 500 μL of digestion buffer containing 1.5 M urea (VWR 97063-798), 50 mM ABC, 10 mM 2-iodoacetamide (VWR 97064-926), and 5 mM TCEP (GoldBio TCEP10). For protein digestion, trypsin protease (Thermo 90057) was reconstituted to 1 μg/μL with 50 mM acetic acid, and 10 μg added to each sample for overnight digestion (16–20 h) on end-over-end rotisserie at room temperature. After digestion, the soluble fraction containing the tryptic peptides was separated from the beads by spinning at 1000 × *g* for 1 min. Samples were then transferred to fresh tube and acidified with 10% TFA to reach a final concentration of 1% TFA. For desalting, C18 SOLA columns (Thermo 03150391) were activated with 500 μL ACN and equilibrated twice with 500 μL 0.1% TFA on a vacuum manifold. Acidified samples were passed through the column twice followed by two washes with 1000 μL 0.1% TFA and one wash with 500 μL 2% ACN/0.1% FA. Finally, peptides were eluted once with 150 μL 80% ACN/0.1% FA and again with 200 μL 80% ACN/0.1% FA. Samples were fully dried by SpeedVac and stored in −80 °C.

### Micro-cell-surface-capture (CSC)

Micro-CSC for samples with cell input of <1e6–5e6 was performed as follows. Labeled cell pellets were lysed in 500 μL buffer containing 2X RIPA (Millipore 20-188), 1X HALT protease inhibitor (Thermo 78430), and 2 mM EDTA. Lysates were sonicated at 1-Hz pulses for 30 s with a probe sonicator and incubated on ice for 10 min. To remove precipitates, samples were spun at 17,200 × *g* for 10 min at 4 °C. To prepare for enrichment of biotinylated proteins, 100 μL of NeutrAvidin slurry (Thermo 29200) were washed three times with 1 mL 2X RIPA/1 mM EDTA buffer in 2-mL chromatography columns (Bio-Rad 7326008) attached to a vacuum manifold (Promega A7231). Clarified lysates were then transferred to the chromatography column and incubated at 4 °C for 120 min on a rotisserie. Beads were washed in the chromatography column with 5 mL each of the following buffers: 1x RIPA/1 mM EDTA, 1X PBS/1M NaCl, and 2 M urea/50 mM ammonium bicarbonate (ABC). P200-StageTips were packed with four C18 disks (3M 14-386-2) and activated with 60 μL methanol, 60 μL 80% acetonitrile (ACN)/0.1% formic acid (FA), and twice with 60 μL 0.1% trifluoroacetic acid (TFA) prior to sealing elution end of stage tip with heat block and transferring the beads to the tip using 100 μL of the pre-diluted digestion buffer (50 mM Tris pH 8.5, 10 mM TCEP, 20 mM IAA, 4M Urea). For protein digestion, 2 μg of reconstituted Trypsin/Lys-C protease (Promega, PRV5073) were added to the sample, which was then wrapped in parafilm and placed on an end-to-end rotisserie at RT for 1–2 h. Samples were then diluted with 200 μL of 50 mM Tris pH 8.5 to activate Tryspin, re-covered with parafilm, and incubated overnight without mixing at RT for digestion. After digestion, 10% TFA was added to each sample to reach a final concentration of 1% TFA. An initial peptide-binding step was performed by spinning the acidified samples at 1000 × *g* prior to desalting three times with 100 μL 0.1% TFA. Peptides were then eluted twice with 50 μL 80% ACN/0.1% FA. Samples were fully dried by SpeedVac and stored in −80 °C.

### SILAC-cell-surface-capture (CSC)

For lenalidomide-resistant cell line analysis using SILAC, all cell lines were grown were cultured in RPMI SILAC media (Thermo) supplemented with Dialyzed FBS for SILAC (Thermo) containing L-[^13^C_6_,^15^N_2_]lysine and L-[^13^C_6_,^15^N_4_] arginine (heavy label; Thermo) or L-[^12^C_6_,^14^N_2_]lysine and L-[^12^C_6_,^14^N_4_]arginine (light label, Thermo) for 5 passages to ensure full incorporation of the isotope labeling on cells. Briefly, 40 × 10^6^ Len sensitive and resistant cells were harvested at 80% confluence, mixed at 1:1 cell count ratio, and subjected to the macro CSC protocol as described above to include all tryptic fragments. All experiments were performed in duplicate in both forward and reverse SILAC labeling scheme such that a total of four biological replicates were analyzed together.

### LC-MS/MS operation

For all analyses except lenalidomide-resistant cell lines and TMT-proteomics, 1 μg of peptides were injected into a Dionex Ultimate 3000 NanoRSLC instrument with 15-cm Acclaim PEPMAP C18 (Thermo) reverse phase column coupled to a Thermo Q Exactive Plus mass spectrometer. A linear gradient from 2.4% Acetonitrile to 40% Acetonitrile in 0.1% Formic Acid over 195 min at flow rate of 0.2 μL/min was employed, with an increase to 80% Acetonitrile for column wash prior to re-equilibration. For MS1 acquisition, spectra were collected in data-dependent top 15 method with full MS scan resolution of 70,000, AGC target was set to 3e6, and maximum IT set to 100 ms. For MS2 acquisition, resolution was set to 17,500, AGC set to 5e4, and maximum IT to 180 ms. For lenalidomide-resistant cell line analysis using SILAC, data were collected on the Q-Exactive Plus in data-dependent mode using a top 20 method with dynamic exclusion of 35 secs and a charge exclusion setting that only sample peptides with a charge of 2, 3, or 4. Survey scans were collected as profile data with a resolution of 140,000 (at 200 *m*/*z*), AGC target of 3E6, maximum injection time of 120 ms, and scan range of 400–1800 *m*/*z*. MS2 scans were collected as centroid data with a resolution of 17,500 (at 200 *m*/*z*), AGC target of 5E4, maximum injection time of 60 ms with normalized collision energy at 27, and an isolation window of 1.5 *m*/*z* with an isolation offset of 0.5 *m*/*z*. For TMT proteomics on primary myeloma and B-cells, 500 ng peptides were injected Easy-Spray reversed phase column (Thermo ES800) on a nanoACQUITY UPLC (Waters) coupled to a Fusion Lumos Mass Spectrometer (Thermo). A 125 min gradient including a linear step from 5% to 30% Acetonitrile over 102 min was used. For MS1 data acquisition, scan range was set to 375–1500 *m*/*z*, AGC target was set to 4e5, and maximum injection time (IT) was set to 50 ms. For MS2 acquisition, and isolation window of 0.7 *m*/*z*, AGC Target 50000, orbitrap resolution of 50 K, maximum injection time of 86 ms, and for HCD 5% stepped collision energy around 35%.

### Proteomic data analysis and quantification

For all analyses except lenalidomide-resistant cell lines, mass spectrometry data was processed in Maxquant^12^ (version 1.6.2.1) with settings as follows: enzyme specificity as trypsin with up to two missed cleavages, PSM/Protein FDR 0.01, cysteine carbidomethylation as fixed modification, methionine oxidation and N-terminal acetylation as variable modifications, minimum peptide length = 7, matching time window 0.7 min, alignment time 20 min, with match between runs, along with other default settings. Data were searched against the Uniprot Swiss-Prot human proteome (downloaded Sept. 3, 2018). proteinGroups files were exported from Maxquant, filtered to remove contaminants, and filtered for proteins with at least one unique peptide for analysis unless specified otherwise. Data analysis was performed in Perseus^66^ and R, and *p*-values used in volcano plots are derived from Welch’s two-sample *t*-tests using the matrixTests package in R. Identified proteins were filtered against curated lists of Uniprot-annotated membrane proteins or those identified in the analysis of Bausch-Fluck et al.^10^ (Supplementary Data 8).

For lenalidomide-resistant cell line analysis using SILAC, peptide search for each individual dataset was performed using ProteinProspector (v5.13.2) against 20203 human proteins (Swiss-prot database, obtained March 5, 2015). Enzyme specificity was set to trypsin with up to two missed cleavage; cysteine carbamidomethyl was set as a fixed modification; methionine oxidation, lysine, and arginine SILAC labels were set as variable modifications; peptide mass tolerance was 6 ppm; fragment ion mass tolerance was 0.4 Da; peptide identification was filtered by peptide score of 0.0005 in Protein Prospector, resulting in a false discovery rate (FDR) of <1% calculated by number of decoy peptides included in the database.

Quantitative data analysis was performed using Skyline software with the MS1 filtering function^67^. Specifically, spectral libraries from forward and reverse SILAC experiments were analyzed together such that MS1 features without an explicit peptide ID would be quantified based on aligned peptide retention time. The first four isotopic peaks of precursor ions were then quantified at 50% FWHM defined by ms1 scanning resolution of 140,000 (at 200 *m*/*z*). The boundary of peptide elution time was determined by default algorithm and the total peak area was used as the peptide quantification value. An isotope dot product of at least 0.8 (as calculated by Skyline) was used to filter out low-quality peptide quantification, and a custom report was generated for further processing and analysis using R. To ensure stringent quantification of the surface proteome, several filters were applied to eliminate low confidence protein identifications. In the tryptic fraction, only peptides with five or more well-quantified peptides were included. Each replicate between individual fractions or reverse SILAC labeling agreed with each other, and forward and reverse SILAC datasets were then combined and reported as median log2 enrichment values. All *p* values reported is Wilcox ranked test for median log2 enrichment SILAC ratio. All data analyses were carried out using R.

### CD53 immunohistochemistry

Decalcified bone marrow core biopsies were obtained from myeloma patients under a UCSF Committee on Human Research-approved protocol (see “Human research participants”, above). Notably, samples were archived, formalin-fixed paraffin-embedded core biopsies and were obtained from distinct patients from those used for flow cytometry analysis on fresh bone marrow aspirates. The electronic medical record was evaluated to identify sequential core biopsies performed on the same patient at both diagnosis and at first relapse. Interpretation was performed by two hematopathologists (S.P. and E.R.) blinded to patient identification and relapse status. Final H-score was averaged between pathologists. CD53 staining was assessed on cells with morphology consistent with plasma cells and CD138+ staining on adjacent tissue sections.

For immunohistochemical staining, tissues were fixed in neutral buffered 10% formalin for 24 to 48 h, dehydrated with graded alcohols, and infiltrated with paraffin wax at 58 degrees in an automated tissue processor. Infiltrated tissue was embedded at 60 °C to produce FFPE blocks. FFPE blocks were sectioned at 4 microns. Immunohistochemical detection on the unstained FFPE tissue sections was performed on Ventana Medical Systems Discovery Ultra Biomarker Automated Slide Preparation System using alkaline epitope conditioning (Ventana/Roche CC1) at 97 °C for 32 min. Recombinant rabbit monoclonal (clone EPR4342(2) directed to CD53 supplied by Abcam (ab134094) used at 1:200 for 32 min at 36 °C. OmniMap anti-Rb HRP (Ventana 760-4311) was used for chromogenic DAB detection (32 min).

### TXNDC11 and CD38 immunofluorescence

AMO1 and MM.1S cells were fixed in 4% PFA in PBS, washed twice with PBS, and incubated with 3% BSA blocking buffer for 1 h at RT. Cells were stained with anti-TXNDC11 (Abcam cat# ab188329) or anti-CD38 (NSJ Bioreagents cat# V3007) at 1:1000 each, follow by incubation with Goat-anti-Rabbit-alexa-488 at 1:500 for TXNDC11 or Goat-anti-mouse-647 at 1:500 for CD38. Prior to imaging, 1:1 volume of DAKO/DAPI was added for nuclear stain. Microscopy was performed on an inverted microscope (Nikon Ti-E) equipped with a spinning-disk confocal and TIRF combined system (Spectral Applied Sciences Diskovery, Ontario, Canada). Cells were imaged via confocal spinning disk microscopy using a 100X Nikon Plan Apo TIRF (NA 1.49) objective on a Nikon Eclipse microscope fitted with an Andor iXon emCCD camera and controlled by Micromanager 2.0.

### Bioinformatic analysis of locking-on and standalone immunotherapy targets

For locking-on analysis, LFQ intensity averages, quartiles, and standard deviation were calculated based on information in Supplementary Data 1. The 14 potential locking-on targets were compared to transcript levels in MMRF_CoMMpass_IA16a_E74GTF_cufflinks_Gene_FPKM dataset.

For standalone immunotherapy targets, the scoring rubric used is described in Supplementary Table 1. For COMPARTMENTS predictions of subcellular localization, the dataset used was “human_compartment_integrated_full.tsv”, available on https://compartments.jensenlab.org/Downloads and accessed on August 27, 2020. The CCLE database used for this analysis was: “CCLE_RNAseq_rsem_genes_tpm_20180929.txt” and accessed on June 25, 2020. Available on https://portals.broadinstitute.org/ccle/data. For the points assignment, a TPM average value was taken and a non-heme cancer cell line average was calculated. Also, the values from each disease were transformed into log2 values (and the non-heme averages). The non-heme diseases were: Bile Duct, Breast, Chondrosarcoma Colorectal, Endometrium, Esophagus, Ewings Sarcoma, Giant cell tumor, Glioma, Kidney, Liver, Lung NSC, Lung small cell, Medulloblastoma, Melanoma, Mesotelioma, Neuroblastoma, Osteosarcoma, Other, Ovary, Pancreas, Prostate, Soft Tissue, Stomach, Thyroid, Upper Aerodigestive, Urinary Tract. The genes where Multiple myeloma were highest expressed compared to the other diseases were selected and compared to the average value in non-heme tissues. For Human Blood Atlas analysis, the database used was “rna_blood_cell_monaco.tsv” available in the Human Protein Atlas portal https://www.proteinatlas.org/about/download (ref. ^68^). Accessed on August 27, 2020. For GTEx analysis, the dataset used was “GTEx_Analysis_2017-06-05_v8_RNASeQCv1.1.9_gene_median_tpm.gct”, accesed on July 16, 2020 and available on https://gtexportal.org/home/datasets. The ESNG gen names were transformed into HUGO nomenclature using gprofiler2 package in RStudio (https://CRAN.R-project.org/package=gprofiler2). We calculated a non-heme tissue average and a heme tissue average. The non-heme tissues were: Adipose Subcutaneous, Adipose Visceral Omentum, Adrenal Gland, Artery Aorta, Artery Coronary, Artery Tibial, Bladder, Brain Amygdala, Brain Caudate basal ganglia, Brain Cerebellar Hemisphere, Brain Cerebellum, Brain Cortex, Brain Frontal Cortex BA9, Brain Hippocampus, Brain Hypothalamus, Brain Nucleus accumbens basal ganglia, Brain Putamen basal ganglia, Brain Spinal cord cervical c1, Brain Substantia nigra, Breast Mammary Tissue, Cells Cultured fibroblasts, Cervix Ectocervix, Cervix Endocervix, Colon Sigmoid, Colon Transverse, Esophagus Gastroesophageal Junction, Esophagus Mucosa, Esophagus Muscularis, Fallopian Tube, Heart Atrial Appendage, Heart Left Ventricle, Kidney Cortex, Kidney Medulla, Liver, Lung, Minor Salivary Gland, Muscle Skeletal, Nerve Tibial, Ovary, Pancreas, Pituitary, Prostate, Skin Not Sun Exposed Suprapubic, Skin Sun Exposed Lower leg, Small Intestine Terminal Ileum, Skin Sun Exposed Lower leg, Small Intestine Terminal Ileum, Stomach, Testis, Thyroid, Uterus, and Vagina. The heme tissues included were Cells-EBV transformed lymphocytes, Spleen, and Whole Blood.

In the end, we merged these 5 datasets, matching by Gene Name, using the controls mentioned above (used across all the process to verify reliability), for 33,654 proteins scored in total. The final dataset were created with all the points assigned across all the dataset, creating a total score which is the sum of the prior points. Then, the proteins were ranked by the total score in a descending manner (see Supplementary Data 2).

### Statistical analyses

Data are presented as mean +/− standard deviation unless specified otherwise. All *t*-tests employed in this study are two-tailed unless otherwise stated. Proteomics significance cutoffs for differentially coloring points in volcano plots (points colored in blue) is absolute value of log2 fold change >= 1 and *p*-value <0.05 unless otherwise stated. RNA-seq was conducted with two biological replicates. For label-free proteomics on cell lines, experiments were conducted in two or three biological replicates except RPMI-8226-BtzR cells. For drug screening and CAR-T cytotoxicity data, technical replicates describe independent wells in an assay plate. The 95% Confidence Intervals (Cis) for IC50s of Bortezomib screens were calculated using GraphPad with “log(inhibitor) vs. normalized response – Variable slope” nonlinear fit method. For box plots shown, unless otherwise stated, upper and lower hinges correspond to 25 and 75 percentiles, upper and lower whiskers extend to highest and lowest values within 1.5* IQR of the hinge, and center line corresponds to the median unless otherwise stated.

### Reporting summary

Further information on research design is available in the Nature Research Reporting Summary linked to this article.

## Supplementary information

Supplementary Information

Description of Additional Supplementary Files

Dataset 1

Dataset 2

Dataset 3

Dataset 4

Dataset 5

Dataset 6

Dataset 7

Dataset 8

Dataset 9

Dataset 10

Reporting Summary

## Data Availability

Proteomic data generated in this study was deposited to ProteomeXchange via the PRIDE database^69,70^ and are available under the accession numbers PXD022482, PXD022553, and PXD032031. Leukemia proteomic data was downloaded from PRIDE, accession number PXD016800. RNA sequencing data generated in this study was uploaded to GEO and are available under the accession number GSE160572. Processed leukemia RNA sequencing data was downloaded from GEO, accession number GSE142447. Figures 2C, D, 3C, 4B and Supplementary Figures 3B–G, 6E, 7G show data from the Multiple Myeloma Research Foundation (MMRF) CoMMpass study, which are available to registered users through the MMRF Researcher Gateway (registration information available at https://mmrf.formstack.com/forms/research_gateway_registration). Supplementary Figures 2A (top), 5A (top), 7J and 11 show data from the Human Protein Atlas, which is available at https://www.proteinatlas.org/. Supplementary Figures 2A (bottom), Fig. 5A (bottom) and 6C show data from GTEx, which are available at https://gtexportal.org/home/. Source data are provided with this paper.

## Source data

Source Data
