# Supplementary material for: The surfaceome of multiple myeloma cells suggests potential immunotherapeutic strategies and protein markers of drug resistance
Source: Nat Commun. 2022 Jul 15;13:4121. doi: 10.1038/s41467-022-31810-6 (PMC9287322; doi:10.1038/s41467-022-31810-6)
Supplement: Supplementary file 1 — Supplementary Information [file 41467_2022_31810_MOESM1_ESM.pdf]

Supplementary Information For:

The surfaceome of multiple myeloma cells suggests potential immunotherapeutic strategies and protein markers of drug resistance

I.D. Ferguson *et al.*

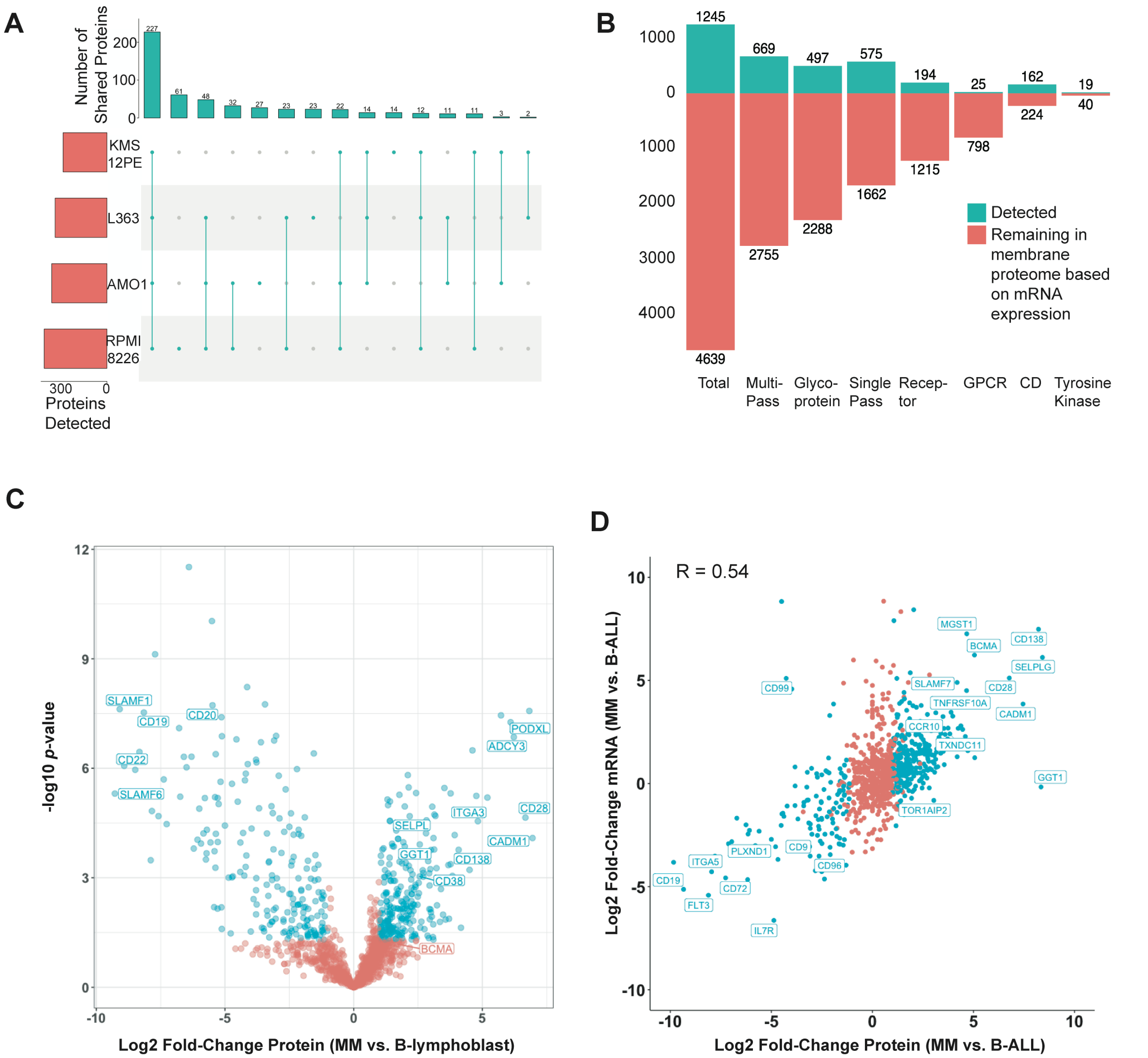
A
Number of Shared Proteins
200
100
0
227
61
48
32
27
23
23
22
14
14
12
11
11
3
2
KMS 12PE
L363
AMO1
RPMI 8226
300
0
Proteins Detected
B
1000
0
1000
2000
3000
4000
1245
669
497
575
194
25
162
19
4639
2755
2288
1662
1215
798
224
40
Detected
Remaining in membrane proteome based on mRNA expression
Total
Multi-Pass
Glyco-protein
Single Pass
Recep-tor
GPCR
CD
Tyrosine Kinase
C
-log10 p-value
Log2 Fold-Change Protein (MM vs. B-lymphoblast)
SLAMF1
CD19
CD20
CD22
SLAMF6
PODXL
ADCY3
CD28
ITGA3
SELPL
CADM1
GGT1
CD138
CD38
BCMA
D
R = 0.54
Log2 Fold-Change mRNA (MM vs. B-ALL)
Log2 Fold-Change Protein (MM vs. B-ALL)
MGST1
BCMA
CD138
SELPLG
CD99
SLAMF7
CD28
TNFRSF10A
CADM1
CCR10
TXNDC11
GGT1
TOR1AIP2
ITGA5
PLXND1
CD9
CD96
CD19
CD72
FLT3
IL7R

Supplementary Figure 1. Additional characterization of the baseline myeloma surfaceome. A. Quantified captured proteins (minimum 2 peptides per protein) filtered based on highest-confidence plasma membrane localization, from ref.[10]. **B.** All genes expressed at mRNA level TPM > 1 in the four analyzed cell lines included (data available at keatslab.org/data-repository, file = "HMCL66_CUFFLINKS_GENE_FPKM.txt") were filtered by Uniprot annotation for different protein classes. We then evaluated which fraction were detected by our cell surface capture experiments. **C.** Analogous to Fig. 1E, we compared our myeloma surface profiles across myeloma lines (AMO1 (n = 3 biological replicates), L363 (n = 2 biological replicates), RPMI-8226 (n = 3 biological replicates), and KMS12PE (n = 3 biological replicates)) to the two B-lymphoblastoid lines examined (ARH-77 (n = 3 biological replicates), and EBV-immortalized normal donor-derived (n = 3 biological replicates)) to identify markers that most-distinguish plasma cells. Significantly-changed proteins noted in blue ($\log_2$-fold change >|1|; $p < 0.05$ by Welch's t-test). Source data in Supplementary Data 1. **D.** RNA-seq data on B-ALL and myeloma cell lines (as in Fig. 1E) was also analyzed for $\log_2$-fold changes between cell types and compared to $\log_2$-fold changes from proteomics (n = 2 biological replicates per cell line). This analysis identifies proteins that may be under post-transcriptional regulation of surface expression, particularly with significant increases in mRNA but no detected change in captured protein. Proteins labeled in blue identical to those found to be significant in volcano plot of Fig. 1E. Pearson correlation and associated p-value of significance shown. Source data in Supplementary Data 4.

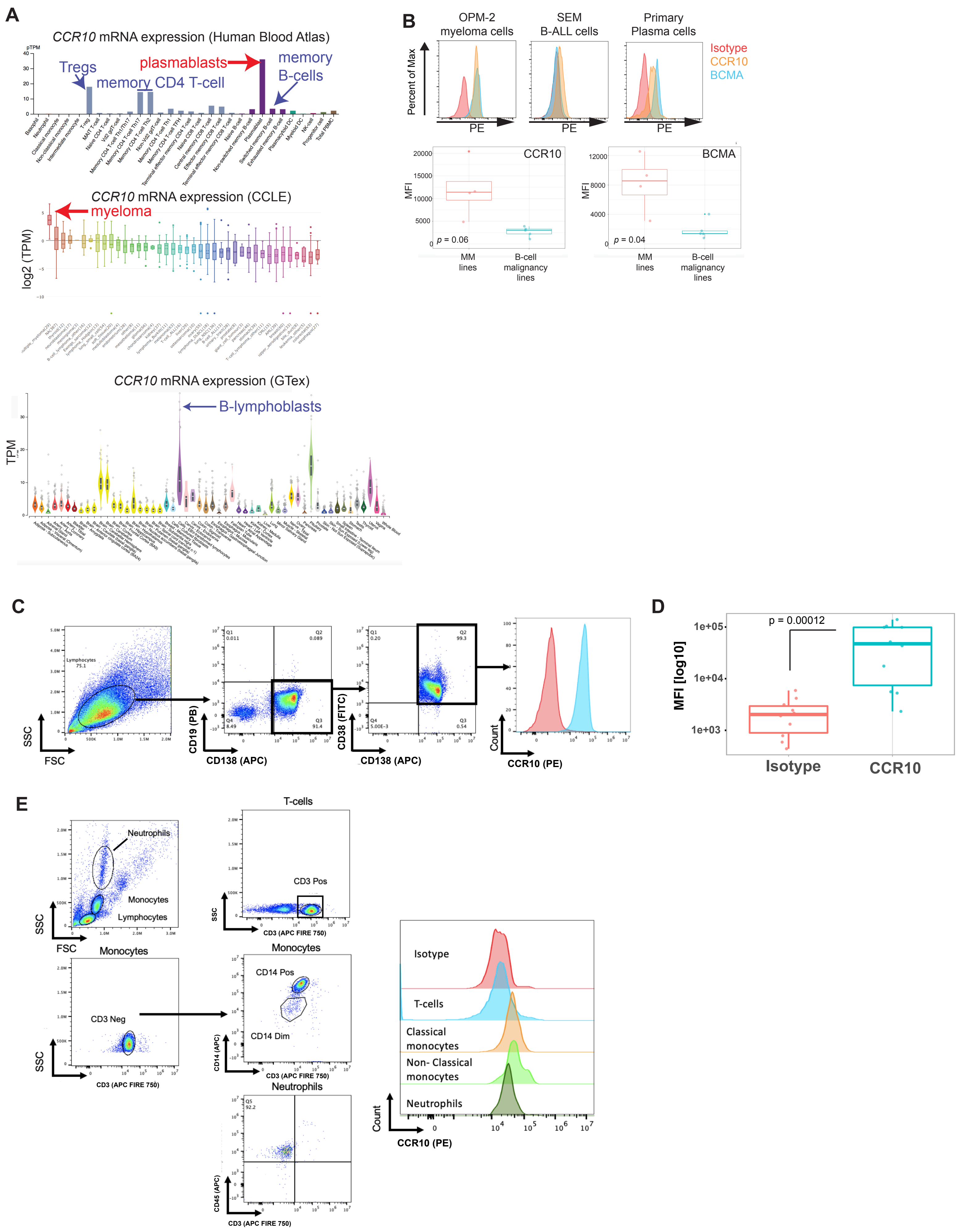
A
CCR10 mRNA expression (Human Blood Atlas)
pTPM
Tregs
memory CD4 T-cell
plasmablasts
memory
B-cells
CCR10 mRNA expression (CCLE)
myeloma
log2 (TPM)
CCR10 mRNA expression (GTex)
B-lymphoblasts
TPM
B
OPM-2
myeloma cells
SEM
B-ALL cells
Primary
Plasma cells
Percent of Max
PE
Isotype
CCR10
BCMA
CCR10
MFI
p = 0.06
MM
lines
B-cell
malignancy
lines
BCMA
p = 0.04
C
Lymphocytes
75.1
SSC
FSC
CD19 (PB)
CD138 (APC)
CD38 (FITC)
Count
CCR10 (PE)
D
p = 0.00012
MFI [log10]
Isotype
CCR10
E
Neutrophils
Monocytes
Lymphocytes
T-cells
CD3 Pos
CD3 (APC FIRE 750)
Monocytes
CD3 Neg
CD14 Pos
CD14 Dim
CD14 (APC)
Neutrophils
CD45 (APC)
Isotype
T-cells
Classical
monocytes
Non- Classical
monocytes
Neutrophils
CCR10 (PE)

Supplementary Figure 2. Additional characterization of CCR10 as a potential myeloma immunotherapy target. A. mRNA transcript data from the Human Blood Atlas (top, n = 30 blood cell types) demonstrates that among hematopoietic cells *CCR10* is most highly expressed in plasmablasts (we note that long-lived plasma cells are not included in this dataset; plasmablasts used as closest proxy). Cancer Cell Line Encyclopedia (CCLE) (middle, n = 1019 cell lines) data supports that on average myeloma cell lines express >20x more *CCR10* mRNA than any other tumor cell type. GTex (bottom) data (V8 release, n = 17382 samples across 54 tissues) does suggest some low level *CCR10* expression in non-hematopoietic tissues; B-lymphoblast expression can be compared to memory B-cell expression in the Human Blood Atlas. Plasmablast expression is therefore expected to be at least 20x higher than other non-hematopoietic tissues, supportive of a therapeutic index. **B.** Representative flow cytometry and quantification confirms significantly higher CCR10 expression on myeloma plasma cells (OPM-2, MM.1S, ANBL-6, AMO-1) than B-cell malignancy cell lines (SEM, RS411 (B-ALL), HBL-1, OCI-LY10, TOLEDO (B-cell lymphoma)), with similar increase as seen for BCMA. Analysis of an initial primary patient plasma cell (CD19-/CD138+/CD38+) specimen confirms CCR10 expression. n = 2 technical replicates per cell line. Box-plots show average MFI between technical replicates for each cell line. **C.** Gating strategy used for analysis in Fig. 2B for primary myeloma cells from patient bone marrow aspirates. CD19-, CD38+, CD138+ cells are selected for CCR10 staining. **D.** Quantification of CCR10 median fluorescence intensity for primary myeloma patient samples (n = 10 patient sample, with two technical replicates per sample and average MFI between technical replicates represented in boxplot) shown in Fig. 2B. p-value by Student's t-test. **E.** Gating strategy and CCR10 expression in T-cells (CD3+), Classical monocytes (CD14+), non-classical monocytes (CD14 dim), and neutrophils (CD45+) isolated from PBMCs. For boxplots in **B** and **D**, upper and lower hinges correspond to 25 and 75 percentiles, upper and lower whiskers extend to highest and lowest values within 1.5* IQR of the hinge, and center line corresponds to the median. For **A, B, D** source data are provided as a Source Data file.

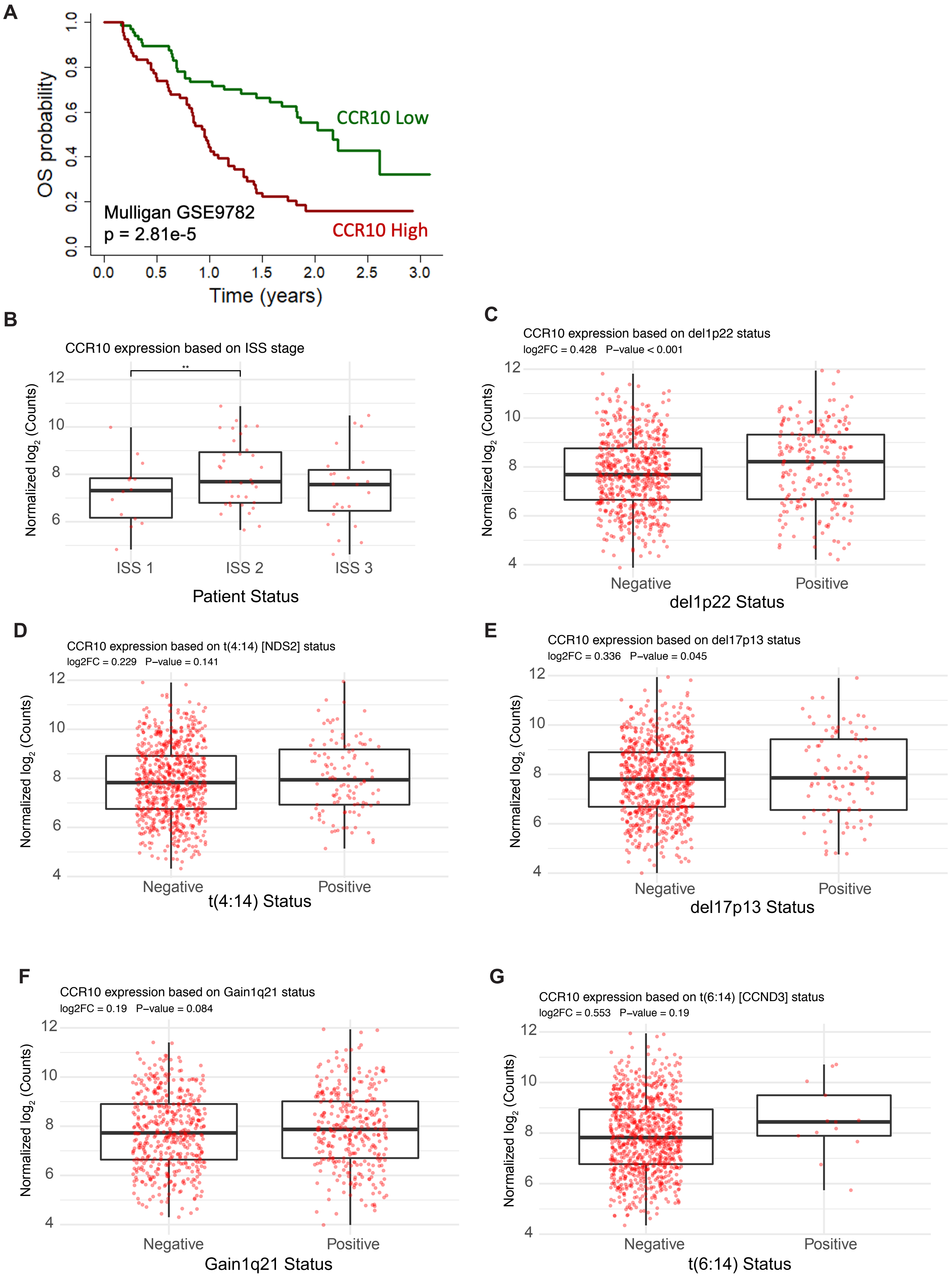
A
OS probability
Time (years)
CCR10 Low
CCR10 High
Mulligan GSE9782
p = 2.81e-5
B
CCR10 expression based on ISS stage
Normalized log2 (Counts)
ISS 1
ISS 2
ISS 3
Patient Status
C
CCR10 expression based on del1p22 status
log2FC = 0.428 P–value < 0.001
Negative
Positive
del1p22 Status
D
CCR10 expression based on t(4:14) [NDS2] status
log2FC = 0.229 P–value = 0.141
t(4:14) Status
E
CCR10 expression based on del17p13 status
log2FC = 0.336 P–value = 0.045
del17p13 Status
F
CCR10 expression based on Gain1q21 status
log2FC = 0.19 P–value = 0.084
Gain1q21 Status
G
CCR10 expression based on t(6:14) [CCND3] status
log2FC = 0.553 P–value = 0.19
t(6:14) Status

Supplementary Figure 3. Additional characterization of CCR10 RNA expression correlates in CoMMpass myeloma clinical dataset. A. Overall survival in Mulligan et al dataset[1], replicating findings from CoMMpass in Fig. 2D. High and Low represent top and bottom 25% of patients by CCR10 gene expression respectively. Number of patients represented in survival plot is 132. *p*-value by log-rank test. **B-G.** CoMMpass myeloma dataset (release = IA19) was parsed by (B) ISS stage (*n* = 85), (C) 1p22 deletion, (D) translocation t(4:14), (E) 17p13 deletion, (F) 1q21 gain, and (G) translocation t(6:14). For C-G, *n* = 854. For all box plots, upper and lower hinges correspond to 25 and 75 percentiles, center line represents the median, and upper and lower whiskers extend to highest and lowest values within 1.5* IQR of the hinge. For B-G, *p*-values by two-sided t-test.

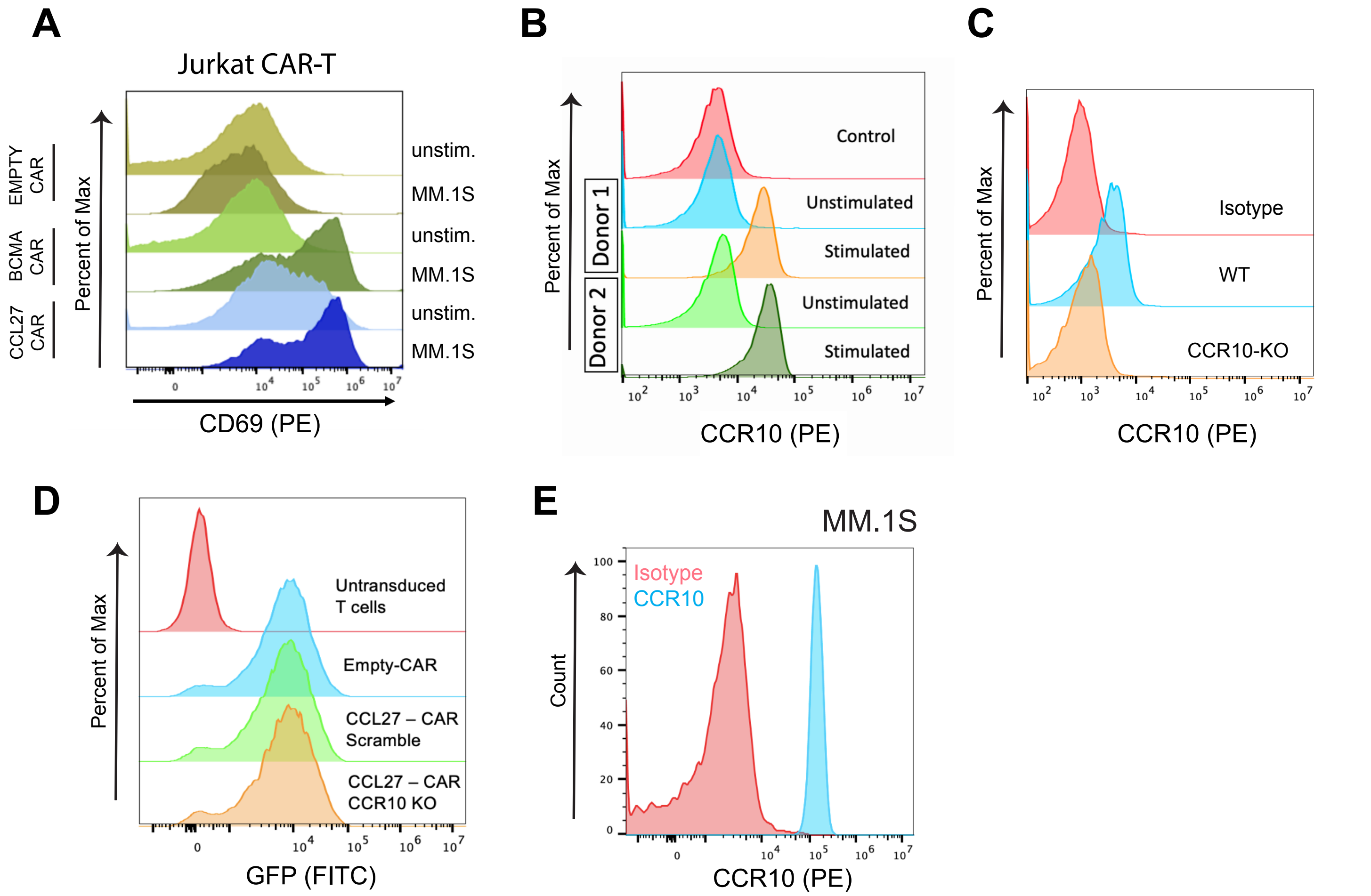
A
Jurkat CAR-T
EMPTY CAR
BCMA CAR
CCL27 CAR
Percent of Max
unstim.
MM.1S
unstim.
MM.1S
unstim.
MM.1S
CD69 (PE)
B
Percent of Max
Donor 1
Donor 2
Control
Unstimulated
Stimulated
Unstimulated
Stimulated
CCR10 (PE)
C
Percent of Max
Isotype
WT
CCR10-KO
CCR10 (PE)
D
Percent of Max
Untransduced T cells
Empty-CAR
CCL27 – CAR Scramble
CCL27 – CAR CCR10 KO
GFP (FITC)
E
MM.1S
Isotype
CCR10
Count
CCR10 (PE)

Supplementary Figure 4. Characterization of CCR10 expression and CCL27 CARs. **A.** Jurkat cells expressing Empty-CAR, BCMA-CAR, or CCL27-CAR were tested for activation when stimulated with MM.1S myeloma cells. Representative of $n = 3$ independent experiments. **B.** CCR10 expression in unstimulated and stimulated T-cells. Representative of $n = 2$ independent experiments. **C.** CCR10 knockout primary T-cells are unable to upregulate CCR10 after bead stimulation. Representative of $n = 2$ technical replicates. **D.** Expression of CAR receptors used for *in-vitro* cytotoxicity (Fig. 2C) measured by GFP. Representative of $n = 2$ technical replicates. **E.** CCR10 expression in MM.1S-luciferase cells by flow cytometry. Representative of $n = 2$ independent experiments.

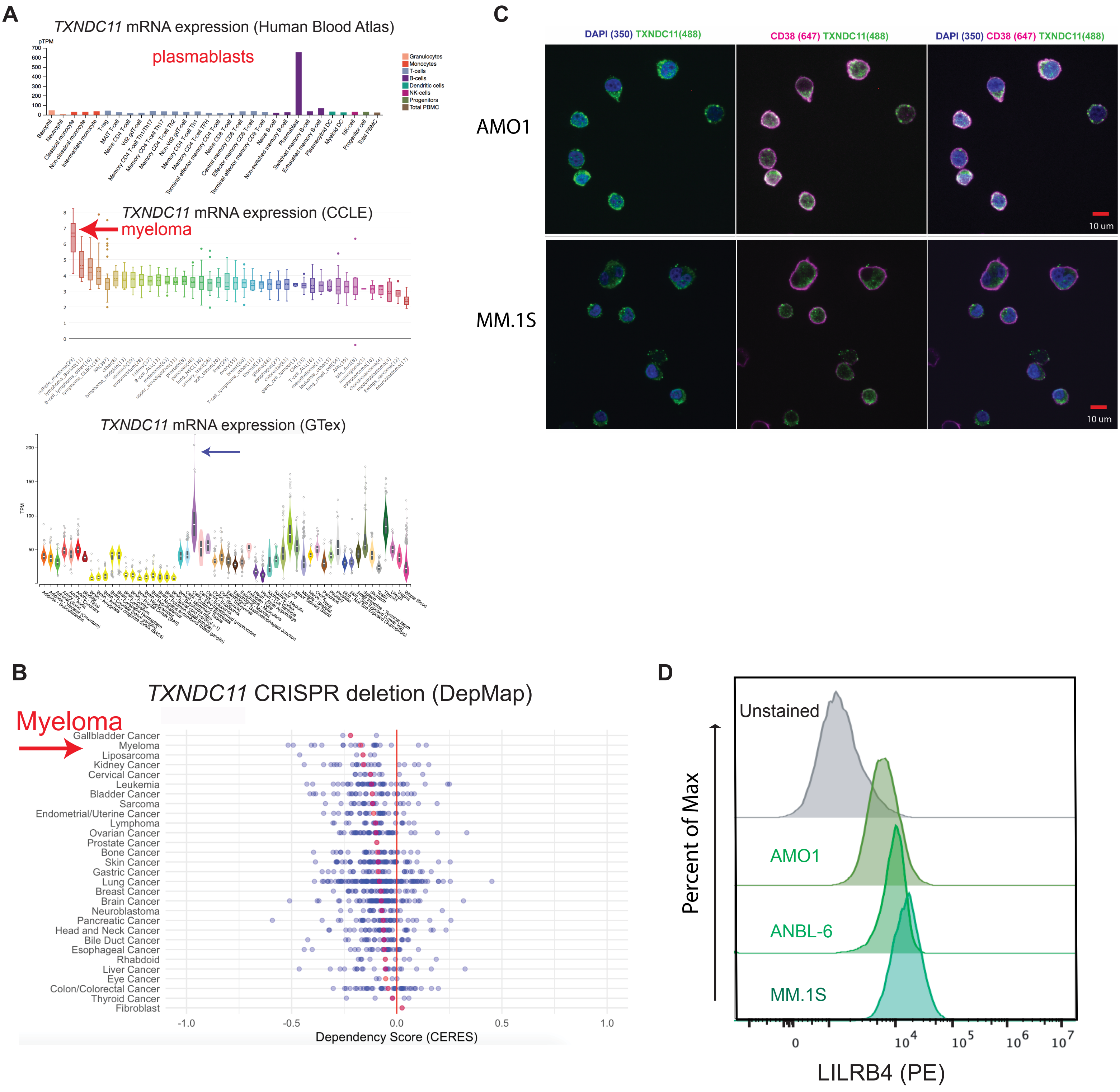
A
TXNDC11 mRNA expression (Human Blood Atlas)
plasmablasts
Granulocytes
Monocytes
T-cells
B-cells
Dendritic cells
NK-cells
Progenitors
Total PBMC
TXNDC11 mRNA expression (CCLE)
myeloma
TXNDC11 mRNA expression (GTex)
C
DAPI (350) TXNDC11(488)
CD38 (647) TXNDC11(488)
DAPI (350) CD38 (647) TXNDC11(488)
AMO1
MM.1S
10 um
10 um
B
TXNDC11 CRISPR deletion (DepMap)
Myeloma
Gallbladder Cancer
Myeloma
Liposarcoma
Kidney Cancer
Cervical Cancer
Leukemia
Bladder Cancer
Sarcoma
Endometrial/Uterine Cancer
Lymphoma
Ovarian Cancer
Prostate Cancer
Bone Cancer
Skin Cancer
Gastric Cancer
Lung Cancer
Breast Cancer
Brain Cancer
Neuroblastoma
Pancreatic Cancer
Head and Neck Cancer
Bile Duct Cancer
Esophageal Cancer
Rhabdoid
Liver Cancer
Eye Cancer
Colon/Colorectal Cancer
Thyroid Cancer
Fibroblast
-1.0
-0.5
0.0
0.5
1.0
Dependency Score (CERES)
D
Unstained
AMO1
ANBL-6
MM.1S
Percent of Max
0
10^4
10^5
10^6
10^7
LILRB4 (PE)

Supplementary Figure 5. Preliminary characterization of TXNDC11 and LILRB4 expression in myeloma. A. mRNA transcript data from the Human Blood Atlas (top, *n* = 30 blood cell types), Cancer Cell Line Encyclopedia (CCLE) (middle, *n* = 1019), and mRNA expression from GTex data (V8 release, *n* = 17382 samples across 54 tissues), illustrating highly enriched *TXNDC11* expression on plasmablasts, highly increased expression in myeloma plasma cells versus any other cancer cell type in the CCLE, and moderate expression in other non-hematopoietic tissues. Upper and lower hinges correspond to 25 and 75 percentiles, upper and lower whiskers extend to highest and lowest values within 1.5* IQR of the hinge, and center line corresponds to the median. **B.** Data from the Cancer Dependency Map (depmap.org; Avana Public 20Q3, *n* = 1019 cell lines) indicate that myeloma cell lines are among the most genetically dependent on CRISPR deletion of this gene, as noted by lowest CERES score when averaged across all included tumor cell lines. For **A-B,** source data are provided as a Source Data file. **C.** Representative confocal microscopy images illustrate that some TXNDC11 is co-localized with canonical cell surface marker CD38 in AMO1 and MM.1S myeloma cells. Two independent experiments were performed and at least five images were obtained for each cell line per experiment. **D.** LILRB4 expression in myeloma cell lines by flow cytometry. Representative of *n* = 2 independent experiments.

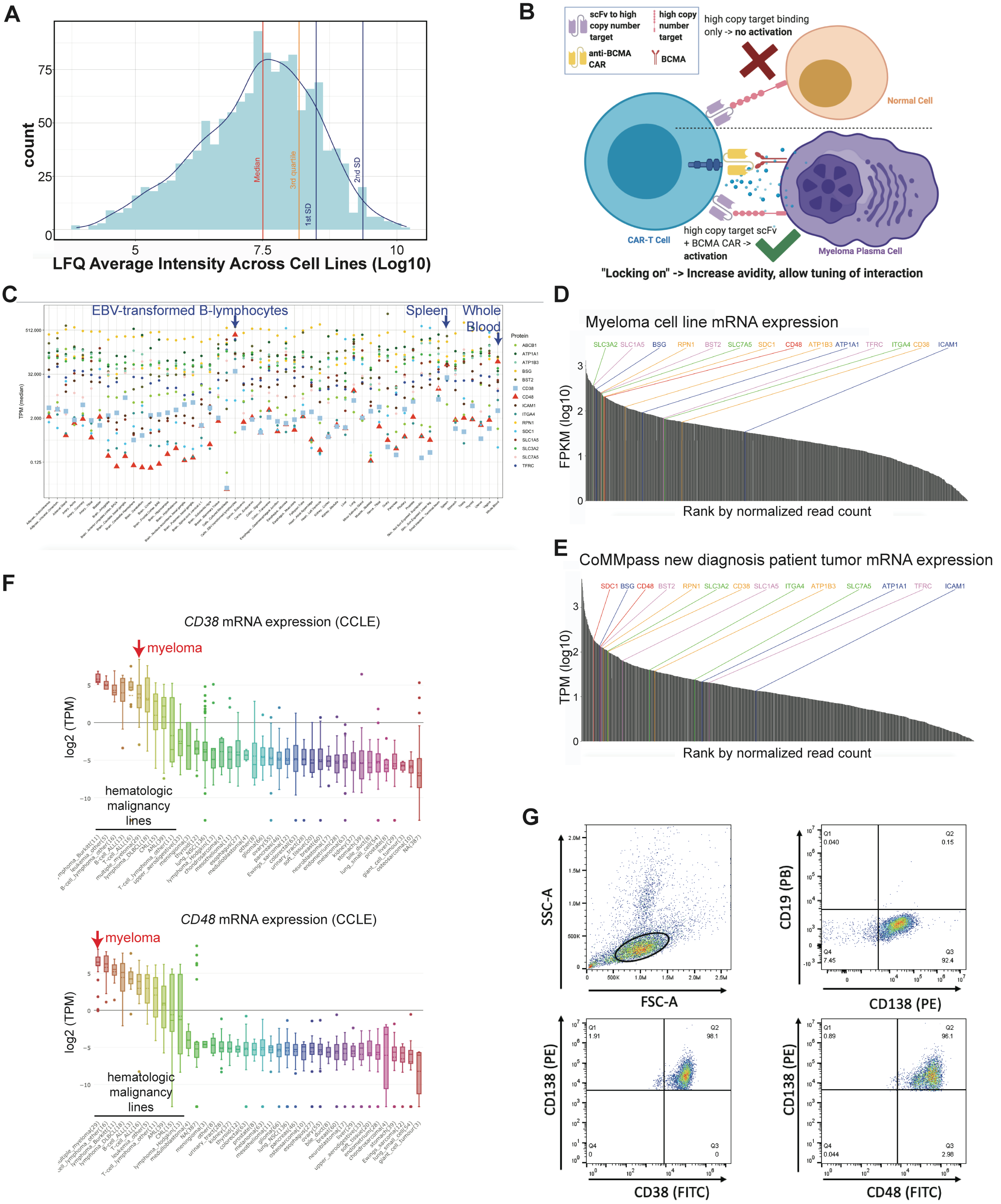
A
count
LFQ Average Intensity Across Cell Lines (Log10)
Median
3rd quartile
1st SD
2nd SD
B
scFv to high copy number target
high copy number target
anti-BCMA CAR
BCMA
high copy target binding only -> no activation
Normal Cell
CAR-T Cell
Myeloma Plasma Cell
high copy target scFv + BCMA CAR -> activation
"Locking on" -> Increase avidity, allow tuning of interaction
C
EBV-transformed B-lymphocytes
Spleen
Whole Blood
TPM (median)
Protein
ABCB1
ATP1A1
ATP1B3
BSG
BST2
CD38
CD48
ICAM1
ITGA4
RPN1
SDC1
SLC1A5
SLC3A2
SLC7A5
TFRC
D
Myeloma cell line mRNA expression
SLC3A2 SLC1A5 BSG RPN1 BST2 SLC7A5 SDC1 CD48 ATP1B3 ATP1A1 TFRC ITGA4 CD38 ICAM1
FPKM (log10)
Rank by normalized read count
E
CoMMpass new diagnosis patient tumor mRNA expression
SDC1 BSG CD48 BST2 RPN1 SLC3A2 CD38 SLC1A5 ITGA4 ATP1B3 SLC7A5 ATP1A1 TFRC ICAM1
TPM (log10)
Rank by normalized read count
F
CD38 mRNA expression (CCLE)
myeloma
log2 (TPM)
hematologic malignancy lines
CD48 mRNA expression (CCLE)
myeloma
log2 (TPM)
hematologic malignancy lines
G
SSC-A
FSC-A
CD19 (PB)
CD138 (PE)
Q1 0.040
Q2 0.15
Q4 7.45
Q3 92.4
CD138 (PE)
CD38 (FITC)
Q1 1.91
Q2 98.1
Q4 0
Q3 0
CD138 (PE)
CD48 (FITC)
Q1 0.89
Q2 96.1
Q4 0.044
Q3 2.98

Supplementary Figure 6. Additional characterization of high abundance surface antigens for a potential "locking on" strategy. A. Distribution of mass spectrometric intensity (label-free quantification (LFQ) from MaxQuant) of all glycoproteins quantified in our proteomic data; intensity is averaged across all four analyzed cell lines. **B.** Schematic illustrating the concept of using a high-abundance, "locking on" antigen to tune CAR-T activation via increased avidity. **C.** Plot of "locking on" candidates (as in Fig. 2D) average transcript expression in GTex (V8 release, *n* = 17382 samples across 54 tissues) suggests that most genes have significant expression in non-hematopoietic tissues, with the exception of CD38 and CD48. **D-E.** mRNA expression of all genes corresponding to proteins identified in our surface proteomic datasets were ranked according to average abundance (measured in TPM) in both cell lines (D) (data available at keatslab.org/data-repository, file = "HMCL66_CUFFLINKS_GENE_FPKM.txt", *n* = 66 myeloma cell lines) and primary CD138+ tumor cells (E) (MMRF CoMMpass release IA16a, *n* = 935 patients). Potential "locking on" candidates are highlighted. **F.** CCLE data highlights that myeloma cell lines express the highest average *CD48* mRNA, whereas several other hematopoietic malignancy cell types express greater *CD38* than myeloma cells. These data also confirm that non-hematopoietic cells do not appear to express these antigens (*n* = 1019). Upper and lower hinges correspond to 25 and 75 percentiles, upper and lower whiskers extend to highest and lowest values within 1.5* IQR of the hinge, and center line corresponds to the median. Source data are provided as a Source Data file**. G.** Illustration of flow cytometry gating strategy to identify primary patient tumor cells for use in absolute quantification of CD38 and CD48. Mononuclear cells in patient bone marrow aspirate were gated on singlet, live lymphocytes in the SSC/FSC plot and then characterized as CD138+/CD19- plasma cells.

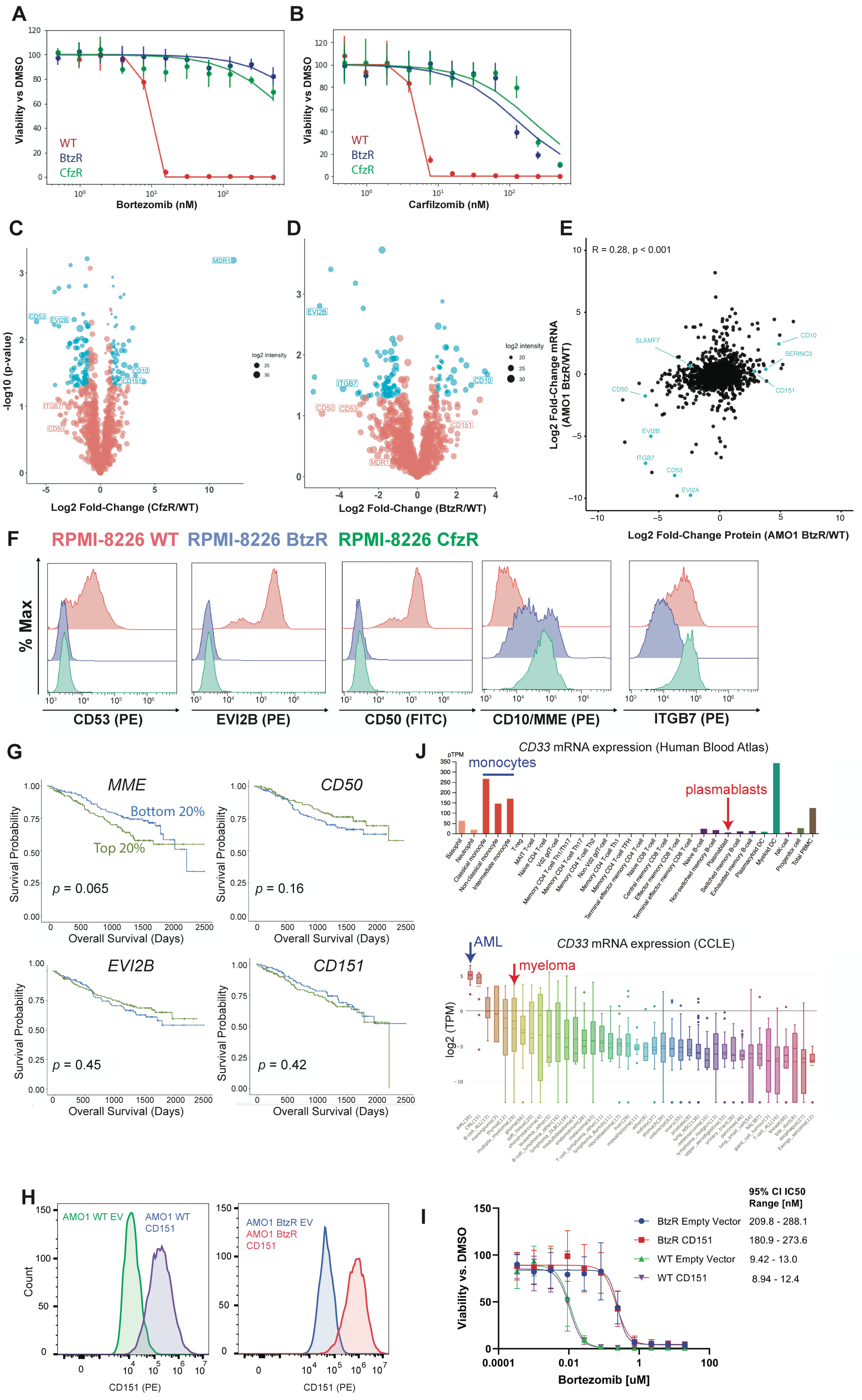
A
B
C
D
E
F
G
H
I
J
Viability vs DMSO
Bortezomib (nM)
Carfilzomib (nM)
WT
BtzR
CfzR
-log10 (p-value)
Log2 Fold-Change (CfzR/WT)
Log2 Fold-Change (BtzR/WT)
R = 0.28, p < 0.001
Log2 Fold-Change mRNA (AMO1 BtzR/WT)
Log2 Fold-Change Protein (AMO1 BtzR/WT)
RPMI-8226 WT RPMI-8226 BtzR RPMI-8226 CfzR
% Max
CD53 (PE)
EVI2B (PE)
CD50 (FITC)
CD10/MME (PE)
ITGB7 (PE)
MME
Bottom 20%
Top 20%
p = 0.065
CD50
p = 0.16
EVI2B
p = 0.45
CD151
p = 0.42
Survival Probability
Overall Survival (Days)
CD33 mRNA expression (Human Blood Atlas)
monocytes
plasmablasts
AML
CD33 mRNA expression (CCLE)
myeloma
log2 (TPM)
AMO1 WT EV
AMO1 WT CD151
AMO1 BtzR EV
AMO1 BtzR CD151
Count
CD151 (PE)
95% CI IC50 Range [nM]
BtzR Empty Vector 209.8 - 288.1
BtzR CD151 180.9 - 273.6
WT Empty Vector 9.42 - 13.0
WT CD151 8.94 - 12.4
Viability vs. DMSO
Bortezomib [uM]

Supplementary Figure 7. Additional characterization of surface biomarker candidates of myeloma drug resistance. A-B. Dose response curves for RPMI-8226 WT, BtzR, and CfzR evolved resistance lines treated with Btz (A) or Cfz (B) for 48 hours. (n = 4 technical replicates). Error bars represent +/- SD. **C-D.** Analogous to Fig. 3A, we display aggregate data of carfilzomib (C) or bortezomib (D) evolved resistant cell lines (CfzR and BtzR, respectively) versus their parental counterpart. Consistent with prior findings[35], we note that MDR1 is by far the most highly-upregulated surface protein in CfzR lines. Significantly-changed proteins noted in blue (log_2-fold change >|1|; $p <$ 0.05). Source Data in Supplementary Data 3. **E.** Surface proteome-transcriptome comparison of BtzR AMO-1 vs. parental AMO-1 cells shows positive but relatively weak correlation (Pearson correlation (R) and associated p-value shown). Blue color used for labeled proteins for ease of point identification**.** Source Data in Supplementary Data 3-4. **F.** Flow cytometry confirms surface proteomic alterations in PI-resistant RPMI-8226 cells as also seen in AMO-1 cells (Fig. 3B). **G.** MMRF CoMMpass (Release IA15, n = 1143 patients) overall survival data for tumor mRNA expression of noted PI-resistance biomarker candidates. To increase potential contrast, the comparison is made between those patient tumors in the top quintile versus bottom quintile of expression. p-value by log-rank test. **H.** Flow cytometry confirms successful CD151 overexpression in AMO1 WT and AMO1 BtzR cells. Representative of n = 2 technical replicates. **I.** AMO1 WT or BtzR cells transduced with CD151 expressing lentivirus or empty vector lentivirus were treated with Bortezomib for 48 hours. n at least 5 technical replicates. Error bars represent +/- SD. **J.** *CD33* is predicted to be expressed at far lower levels on plasmablasts (used as a proxy for plasma cells) than on normal myeloid lineage cells per data in the Human Blood Atlas (top, n = 30 blood cell types), and myeloma cell lines express far less CD33 than AML cell lines in the CCLE (bottom) (n = 1019 cancer cell lines). For boxplots, upper and lower hinges correspond to 25 and 75 percentiles, upper and lower whiskers extend to highest and lowest values within 1.5* IQR of the hinge, and center line corresponds to the median. For **A-B, I, J** source data are provided as a Source Data file**.**

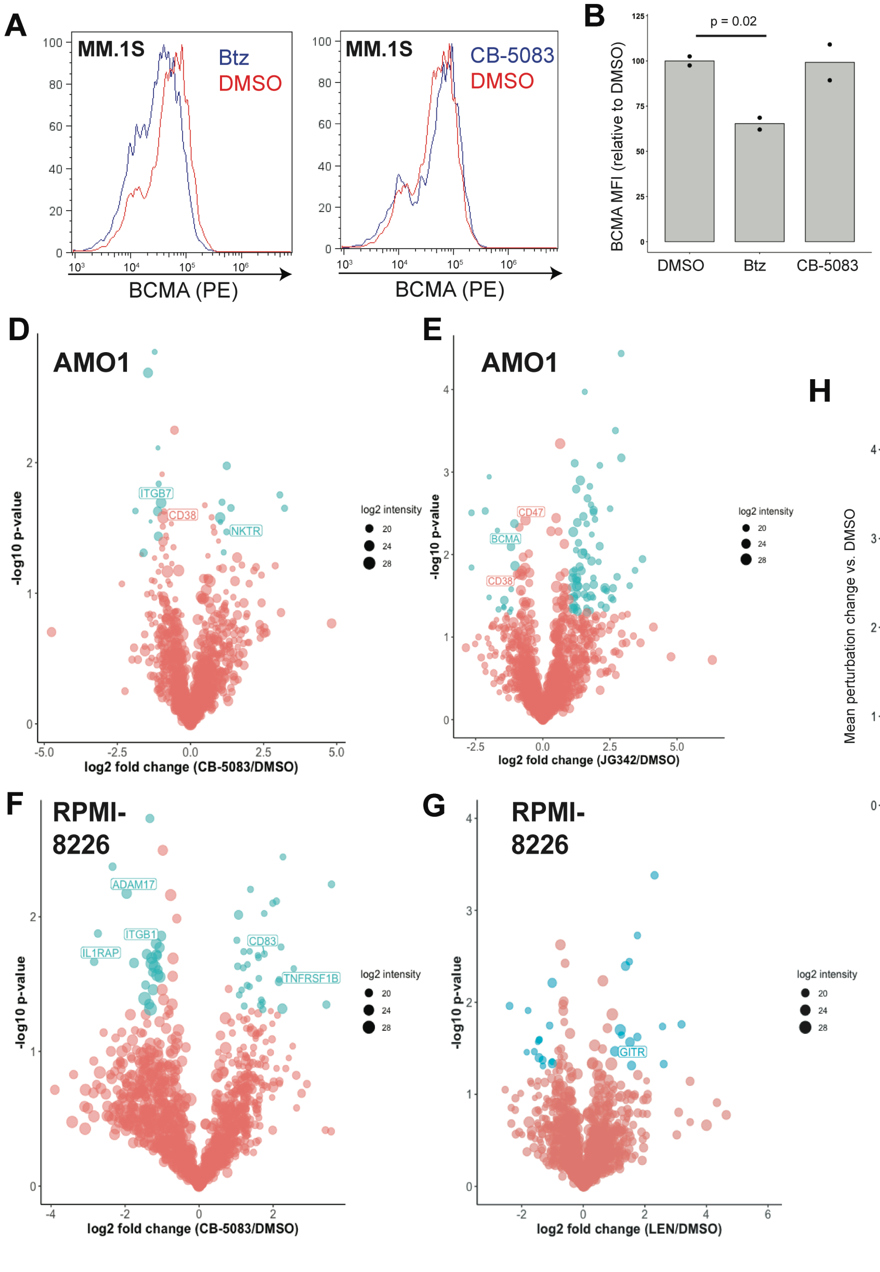
A
MM.1S
Btz
DMSO
BCMA (PE)
MM.1S
CB-5083
DMSO
BCMA (PE)
B
p = 0.02
BCMA MFI (relative to DMSO)
DMSO
Btz
CB-5083
C
T cells
Untransduced
Transduced
and Enriched
Unit Area
MUC1-CAR (FITC)
D
AMO1
ITGB7
CD38
NKTR
-log10 p-value
log2 fold change (CB-5083/DMSO)
log2 intensity
E
AMO1
CD47
BCMA
CD38
-log10 p-value
log2 fold change (JG342/DMSO)
log2 intensity
H
Mean perturbation change vs. DMSO
ITGB6
CD53
BCMA
CD151
EVI2B
SDC1
CD38
CD33
CD48
ITGB7
PTPRC
TXNDC11
CCR10
CD50
Protein Rank
F
RPMI-
8226
ADAM17
ITGB1
IL1RAP
CD83
TNFRSF1B
-log10 p-value
log2 fold change (CB-5083/DMSO)
log2 intensity
G
RPMI-
8226
GITR
-log10 p-value
log2 fold change (LEN/DMSO)
log2 intensity

Supplementary Figure 8. Probing myeloma surfaceome effects of acute drug treatment. A. MM.1S cells treated with 2.5 nM Btz for 48 hours show a decrease in surface BCMA as measured by flow cytometry, while treatment with 250nM CB-5083 for 48 hours shows no change. Representative data of $n = 2$ biological replicates. **B.** Quantification of results in (A) using mean fluorescence intensity ($n = 2$ biological replicates). p-value by Student's t-test. Source data are provided as a Source Data file. **C.** Anti-MUC1 CAR-T construct expression validated by flow cytometry. Representative of $n = 2$ technical replicates. **D-G.** Profiling of AMO1 cells treated with 250 nM CB-5083 for 48 hours (D), or 750 nM JG-342 (E) for 48 hours and RPMI-8226 cells treated with 50 μM Len for 48 hours (F), or 300 nM CB-5083 for 48 hours (G) shows remodeling of the surface proteome ($n = 3$ biological replicates). Source Data in Supplementary Data 5. **H.** Mean of protein absolute value change for 48 hour perturbations vs. DMSO for RPMI (Lenalidomide, Bortezomib, CB-5083) and AMO1 (Lenalidomide, CB-5083, JG342). Proteins on the right side of the distribution show the greatest variability across drug treatments while those on the left side show the least variability.

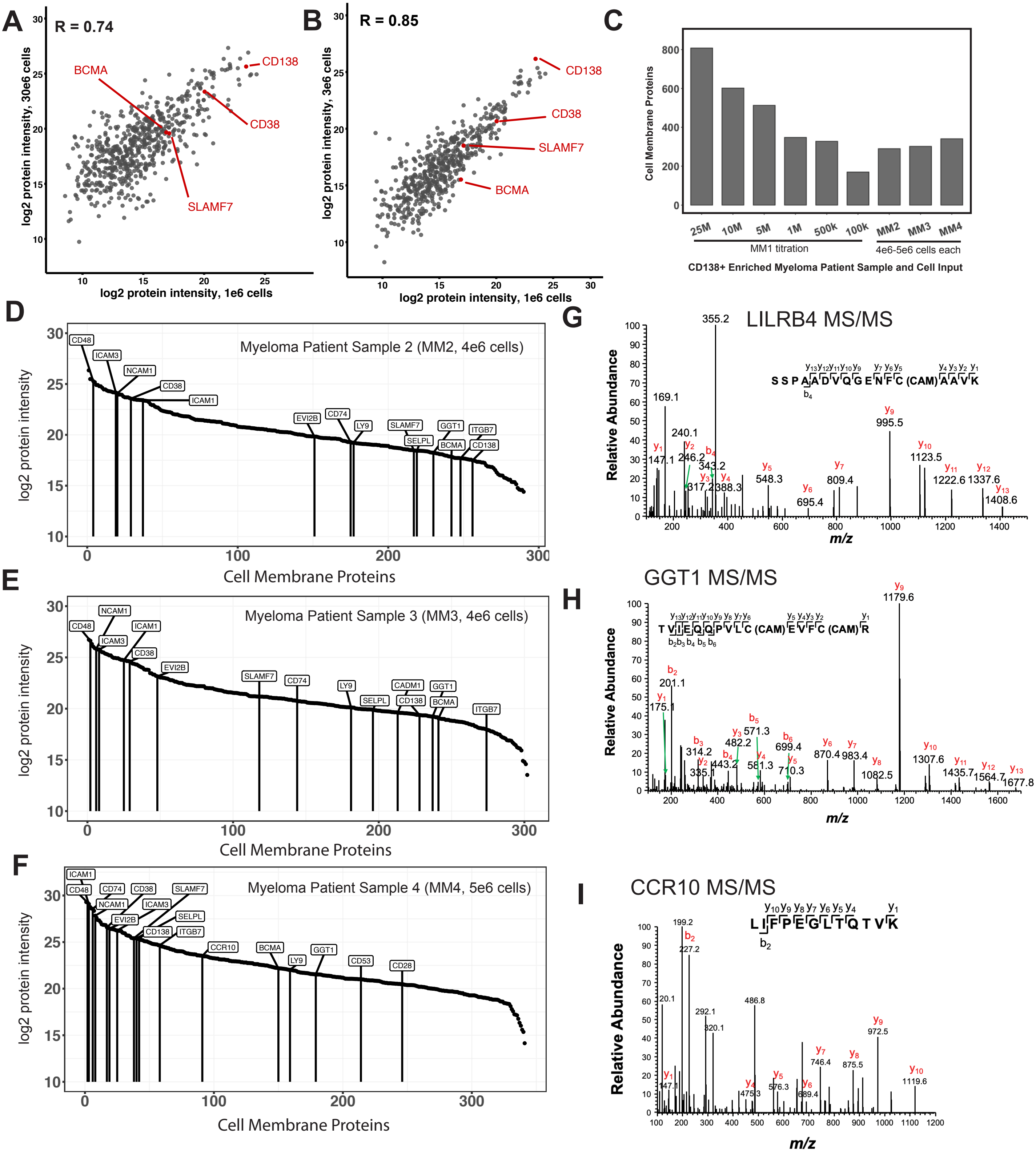
A
R = 0.74
BCMA
CD138
CD38
SLAMF7
log2 protein intensity, 30e6 cells
log2 protein intensity, 1e6 cells
B
R = 0.85
CD138
CD38
SLAMF7
BCMA
log2 protein intensity, 3e6 cells
log2 protein intensity, 1e6 cells
C
Cell Membrane Proteins
25M
10M
5M
1M
500k
100k
MM2
MM3
MM4
MM1 titration
4e6-5e6 cells each
CD138+ Enriched Myeloma Patient Sample and Cell Input
D
Myeloma Patient Sample 2 (MM2, 4e6 cells)
CD48
ICAM3
NCAM1
CD38
ICAM1
EVI2B
CD74
LY9
SLAMF7
SELPL
GGT1
BCMA
ITGB7
CD138
log2 protein intensity
Cell Membrane Proteins
E
Myeloma Patient Sample 3 (MM3, 4e6 cells)
NCAM1
CD48
ICAM3
ICAM1
CD38
EVI2B
SLAMF7
CD74
LY9
SELPL
CADM1
CD138
GGT1
BCMA
ITGB7
log2 protein intensity
Cell Membrane Proteins
F
Myeloma Patient Sample 4 (MM4, 5e6 cells)
ICAM1
CD48
CD74
CD38
SLAMF7
NCAM1
ICAM3
EVI2B
SELPL
CD138
ITGB7
CCR10
BCMA
LY9
GGT1
CD53
CD28
log2 protein intensity
Cell Membrane Proteins
G
LILRB4 MS/MS
S S P A A D V Q G E N F C (CAM) A A V K
Relative Abundance
m/z
H
GGT1 MS/MS
T V I E Q Q P V L C (CAM) E V F C (CAM) R
Relative Abundance
m/z
I
CCR10 MS/MS
L I F P E G L T Q T V K
Relative Abundance
m/z

Supplementary Figure 9. “Micro” method application to myeloma cell lines and primary samples. A-B. Comparison of micro scale proteomics for membrane associated proteins for 1e6 vs. 30e6 and 1e6 vs. 3e6 AMO1 cell input**.** Pearson *R* shown. Source Data in Supplementary Data 9. **C.** Total number of cell membrane-associated proteins identified using the “micro” method at various cell inputs, on CD138+ tumor cells isolated from four relapsed/refractory myeloma patients. Cell input from patient MM1 was titrated using micro protocol. (As in **Fig. 6E,** source data are provided as a Source Data file**) D-F.** Cell membrane associated proteins identified with micro proteomics performed on CD138+ myeloma cells isolated from primary patient samples. Source Data in Supplementary Data 7. **G-I.** MS/MS peptide spectra for LILRB4 (G), GGT1 (H), and CCR10 (I) peptide identified in primary myeloma patient samples.

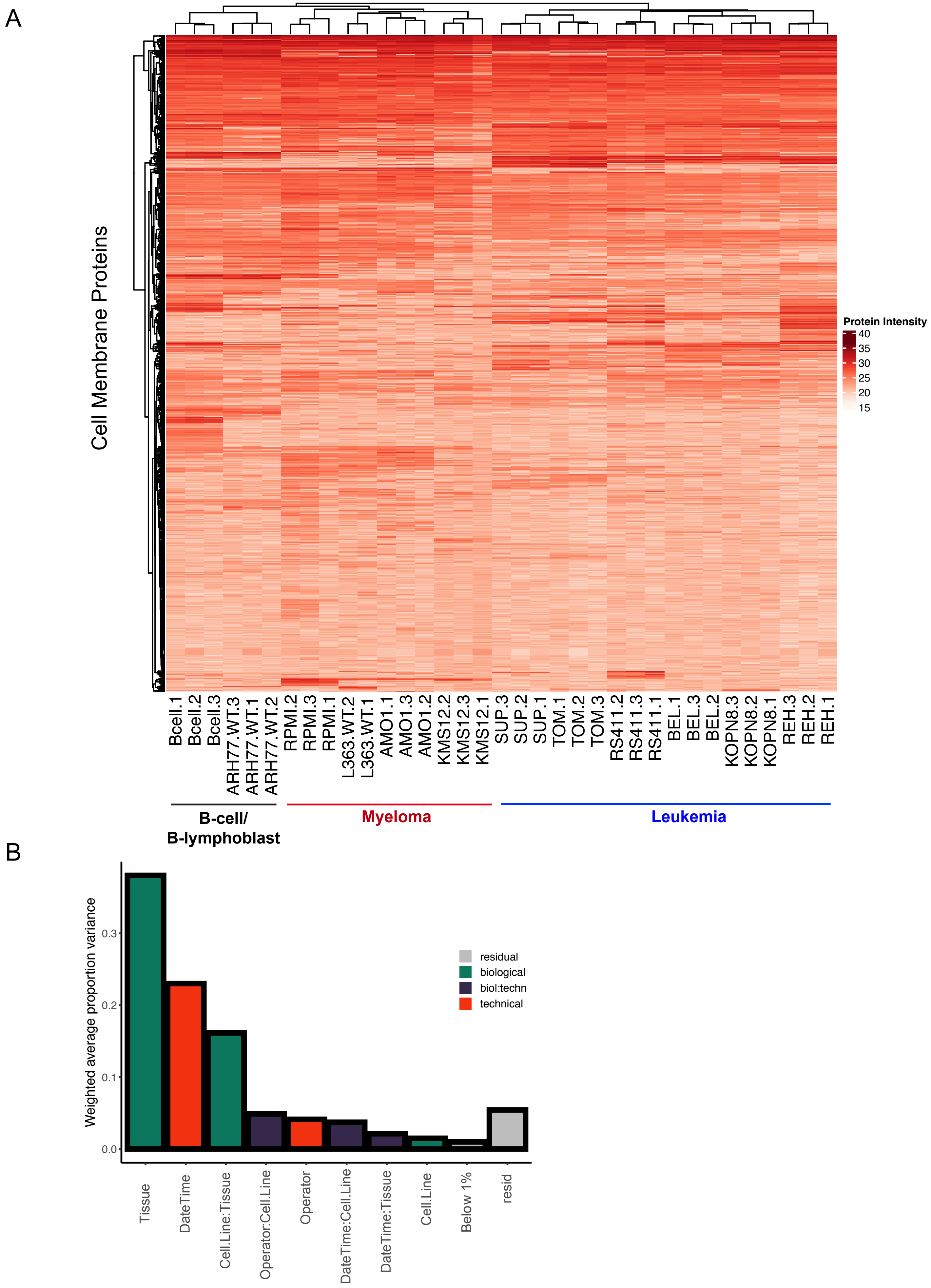
A
Cell Membrane Proteins
Protein Intensity
40
35
30
25
20
15
Bcell.1
Bcell.2
Bcell.3
ARH77.WT.3
ARH77.WT.1
ARH77.WT.2
RPMI.2
RPMI.3
RPMI.1
L363.WT.2
L363.WT.1
AMO1.1
AMO1.3
AMO1.2
KMS12.2
KMS12.3
KMS12.1
SUP.3
SUP.2
SUP.1
TOM.1
TOM.2
TOM.3
RS411.2
RS411.3
RS411.1
BEL.1
BEL.3
BEL.2
KOPN8.3
KOPN8.2
KOPN8.1
REH.3
REH.2
REH.1
B-cell/
B-lymphoblast
Myeloma
Leukemia
B
Weighted average proportion variance
0.0
0.1
0.2
0.3
residual
biological
biol:techn
technical
Tissue
DateTime
Cell.Line:Tissue
Operator:Cell.Line
Operator
DateTime:Cell.Line
DateTime:Tissue
Cell.Line
Below 1%
resid

Supplementary Figure 10. Batch effects in cell surface proteomic datasets. A. Hierarchical clustering of cell surface proteomic samples from **Fig. 1D**. Source Data in Supplementary Data 1. **B.** Quantification of batch effects in cell surface proteomic data from **Fig. 1D.** using proBatch[3]. Log_2 transformed protein intensities without imputation were quantile normalized prior to principal variance component analysis. Source data are provided as a Source Data file.

A

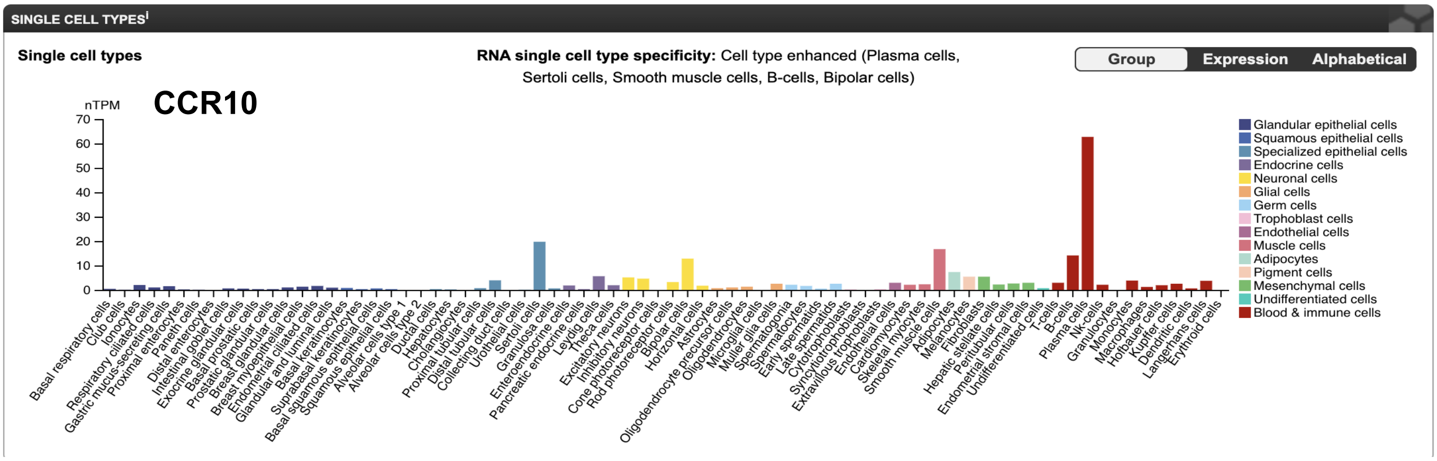
SINGLE CELL TYPES
Single cell types
RNA single cell type specificity: Cell type enhanced (Plasma cells, Sertoli cells, Smooth muscle cells, B-cells, Bipolar cells)
Group
Expression
Alphabetical
nTPM
CCR10
Glandular epithelial cells
Squamous epithelial cells
Specialized epithelial cells
Endocrine cells
Neuronal cells
Glial cells
Germ cells
Trophoblast cells
Endothelial cells
Muscle cells
Adipocytes
Pigment cells
Mesenchymal cells
Undifferentiated cells
Blood & immune cells

B

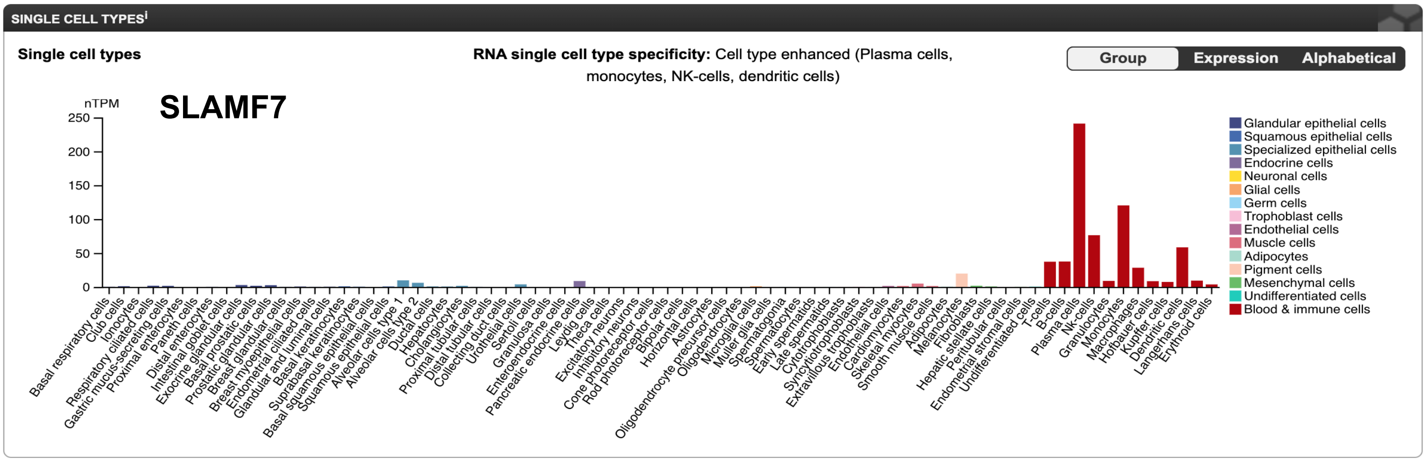
SINGLE CELL TYPES
Single cell types
RNA single cell type specificity: Cell type enhanced (Plasma cells, monocytes, NK-cells, dendritic cells)
Group
Expression
Alphabetical
nTPM
SLAMF7
Glandular epithelial cells
Squamous epithelial cells
Specialized epithelial cells
Endocrine cells
Neuronal cells
Glial cells
Germ cells
Trophoblast cells
Endothelial cells
Muscle cells
Adipocytes
Pigment cells
Mesenchymal cells
Undifferentiated cells
Blood & immune cells

C

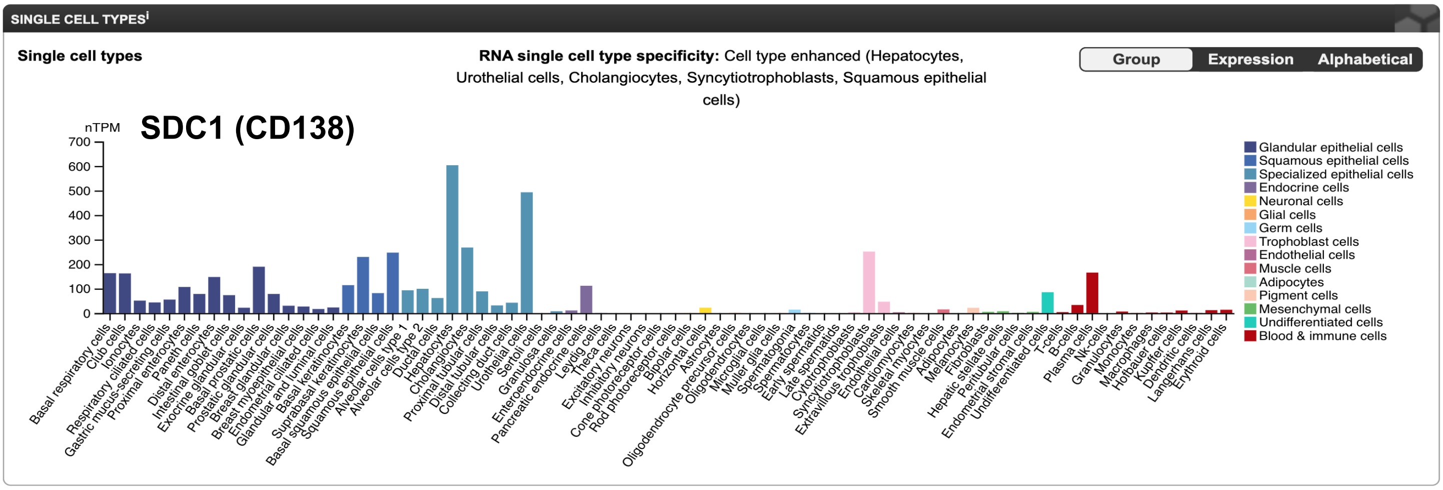
SINGLE CELL TYPES
Single cell types
RNA single cell type specificity: Cell type enhanced (Hepatocytes, Urothelial cells, Cholangiocytes, Syncytiotrophoblasts, Squamous epithelial cells)
Group
Expression
Alphabetical
nTPM
SDC1 (CD138)
Glandular epithelial cells
Squamous epithelial cells
Specialized epithelial cells
Endocrine cells
Neuronal cells
Glial cells
Germ cells
Trophoblast cells
Endothelial cells
Muscle cells
Adipocytes
Pigment cells
Mesenchymal cells
Undifferentiated cells
Blood & immune cells

Supplementary Figure 11. Cell type specificity of CCR10 relative to other myeloma immunotherapy target and marker proteins. A-C. Single-cell RNA-sequencing based cell type expression data from the Human Protein Atlas for **(A)** CCR10, **(B)**, SLAMF7, and **(C)** SDC1/CD138, obtained from web-portal at https://www.proteinatlas.org/.

SUPPLEMENTARY TABLE 1

Dataset	5 points	4 points	3 points	2 points	1 point	0 points
**Myeloma surface proteomics (this study)**			Identified in aggregate proteomic dataset*			Not identified in aggregate proteomic dataset
**COMPARTMENTS (subcellular localization prediction)**			Highest-confidence prediction at plasma membrane		Low-confidence prediction of plasma membrane localization	No predicted localization to plasma membrane
**Cancer Cell Line Encyclopedia**	Highest expression in myeloma cells, with >16-fold (4-fold on log2 scale) greater average expression than average in non-hematopoietic tumor types**		Highest expression in myeloma cells, but not meeting >16-fold threshold	Myeloma cell lines within top 5 most highly-expressing tumor cell types	Expressed in myeloma cells but not highly compared to other tumor types	Not expressed in myeloma cells
**Human Blood Atlas[2]**		At least 10-fold higher expression in plasmablasts*** than any NK cells, T-cell, or myeloid cell type		Highest expression in plasmablasts but not meeting >10-fold criteria		Expressed but no enrichment in plasmablasts vs. other hematopoietic cells
**Genotype Tissue Expression (GTex) project**		Average TPM in non-hematopoietic tissues**** <5		Average TPM in non-hematopoietic tissues<50		Average TPM in non-hematopoietic tissues >50

Supplementary Table 1. Scoring rubric to determine potential myeloma surface antigens for immunotherapeutic targeting. Assigned points based on perceived subjective importance to success of an immunotherapeutic strategy versus a specific antigen. Maximum possible score = 19.

*Aggregate dataset includes cell surface capture proteomics on wild-type (RPMI-8226, AMO-1, L363, and KMS12-PE cells.

**Non-hematopoietic cell types include all except "multiple_myeloma", "B-cell_lymphoma_other", "lymphoma_Burkitt", "lymphoma_DLBCL", "B-cell_ALL", "T-cell_ALL", "leukemia_other", "T-cell_lymphoma_other", "AML", "CML", "lymphoma_Hodgkin".

***plasmablasts are used as a proxy for plasma cells as long-lived plasma cells are not analyzed as part of this dataset

****non-hematopoietic tissues include all except "Cells – EBV-transformed lymphocytes", "spleen", and "whole blood".

SUPPLEMENTARY TABLE 2

**Primary sample profiling by flow cytometry**				
Sex	*M*	*F*		
n	**7**	**6**		
Age (Years)	*50-60*	*60-70*	*70-80*	*>80*
n	**2**	**7**	**3**	**1**
Diagnosis	*Myeloma*	*AL Amyloidosis*		
n	**12**	**1**		
Prior Treatment	*No prior treatment*	*Relapsed/refractory*		
n	**4**	**9**		

**Cell Surface Proteomics**				
Sex	*M*	*F*		
n	**3**	**1**		
Age (Years)	*50-60*	*60-70*	*70-80*	*>80*
n	**2**	**1**	**1**	**0**
Diagnosis	*Myeloma*	*AL Amyloidosis*		
n	**4**	**0**		
Prior Treatment	*No prior treatment*	*Relapsed/refractory*		
n	**0**	**4**		

**Immunohistochemistry**				
Sex	*M*	*F*		
n	**7**	**6**		
Age (Years)	*50-60*	*60-70*	*70-80*	*>80*
n	**4**	**8**	**1**	**0**
Age difference (Years) between pre- and post-PI bone marrow	*0*	*1*	*2*	*>2*
n	**6**	**4**	**2**	**1**
Diagnosis	*Myeloma*	*AL Amyloidosis*		
n	**13**	**0**		

Supplementary Table 2. Primary Patient Sample Information. Aggregated primary patient sample information for flow cytometry, cell surface proteomics, and immunohistochemistry (IHC) analyses.

Supplementary References

1. Mulligan, G. *et al.* Gene expression profiling and correlation with outcome in clinical trials of the proteasome inhibitor bortezomib. *Blood* **109**, 3177–3188 (2007).

2. Monaco, G. *et al.* RNA-seq signatures normalized by mRNA abundance allow absolute deconvolution of human immune cell types. *Cell Rep.* **26**, 1627-1640.e7 (2019).

3. Cuklina, J. Computational challenges in biomarker discovery from high-throughput proteomic data. Ph.D. thesis, ETH Zurich (2018).
