# Supplementary material for: The surfaceome of multiple myeloma cells suggests potential immunotherapeutic strategies and protein markers of drug resistance
Source: Nat Commun. 2022 Jul 15;13:4121. doi: 10.1038/s41467-022-31810-6 (PMC9287322; doi:10.1038/s41467-022-31810-6)
Supplement: Supplementary file 2 — Description of Additional Supplementary Files [file 41467_2022_31810_MOESM2_ESM.docx]

File Name: Supplementary Data 1

Description: Mass spectrometry on Myeloma, B-lymphoblast, and Leukemia cell lines.

File Name: Supplementary Data 2

Description: Output of ranking metrics for myeloma immunotherapy targets

File Name: Supplementary Data 3

Mass spectrometry on Proteasome-inhibitor resistant and WT Myeloma cell lines.

File Name: Supplementary Data 4

RNA sequencing on Proteasome-inhibitor resistant and WT Myeloma cell lines.

File Name: Supplementary Data 5

Description: Mass spectrometry on Myeloma cell lines treated with small molecule inhibitors.

File Name: Supplementary Data 6

Description: Mass Spectrometry on Lenalidomide resistant and WT MM cell lines.

File Name: Supplementary Data 7

Description: Mass spectrometry on primary patient samples using “micro” method.

File Name: Supplementary Data 8

Description: Membrane Protein lists use for filtering and data analysis.

File Name: Supplementary Data 9

Description: AMO1 WT and BtzR Micro Proteomics.

File Name: Supplementary Data 10

Description: Primary Myeloma and B-cell Micro TMT Proteomics.
