# Supplementary material for: The surfaceome of multiple myeloma cells suggests potential immunotherapeutic strategies and protein markers of drug resistance
Source: Nat Commun. 2022 Jul 15;13:4121. doi: 10.1038/s41467-022-31810-6 (PMC9287322; doi:10.1038/s41467-022-31810-6)
Supplement: Supplementary file 13 — Reporting Summary [file 41467_2022_31810_MOESM13_ESM.pdf]

nature research

Corresponding author(s): Wiita, Arun P.

Last updated by author(s): May 31, 2022

Reporting Summary

Nature Research wishes to improve the reproducibility of the work that we publish. This form provides structure for consistency and transparency in reporting. For further information on Nature Research policies, see our Editorial Policies and the Editorial Policy Checklist.

Statistics

For all statistical analyses, confirm that the following items are present in the figure legend, table legend, main text, or Methods section.

n/a	Confirmed	
☐	☒	The exact sample size (*n*) for each experimental group/condition, given as a discrete number and unit of measurement
☐	☒	A statement on whether measurements were taken from distinct samples or whether the same sample was measured repeatedly
☐	☒	The statistical test(s) used AND whether they are one- or two-sided<br>*Only common tests should be described solely by name; describe more complex techniques in the Methods section.*
☒	☐	A description of all covariates tested
☐	☒	A description of any assumptions or corrections, such as tests of normality and adjustment for multiple comparisons
☐	☒	A full description of the statistical parameters including central tendency (e.g. means) or other basic estimates (e.g. regression coefficient) AND variation (e.g. standard deviation) or associated estimates of uncertainty (e.g. confidence intervals)
☐	☒	For null hypothesis testing, the test statistic (e.g. *F*, *t*, *r*) with confidence intervals, effect sizes, degrees of freedom and *P* value noted<br>*Give P values as exact values whenever suitable.*
☒	☐	For Bayesian analysis, information on the choice of priors and Markov chain Monte Carlo settings
☒	☐	For hierarchical and complex designs, identification of the appropriate level for tests and full reporting of outcomes
☐	☒	Estimates of effect sizes (e.g. Cohen's *d*, Pearson's *r*), indicating how they were calculated

Our web collection on statistics for biologists contains articles on many of the points above.

Software and code

Policy information about availability of computer code

Data collection	No specific software was used for data collection.
Data analysis	Flow cytometry: FlowJo v. 10.8 or 8.8.6. Mass spectrometry: MaxQuant v.1.6.2.1. Plotting and statistics: R v.3.6

For manuscripts utilizing custom algorithms or software that are central to the research but not yet described in published literature, software must be made available to editors and reviewers. We strongly encourage code deposition in a community repository (e.g. GitHub). See the Nature Research guidelines for submitting code & software for further information.

Data

Policy information about availability of data

All manuscripts must include a data availability statement. This statement should provide the following information, where applicable:
- Accession codes, unique identifiers, or web links for publicly available datasets
- A list of figures that have associated raw data
- A description of any restrictions on data availability

Proteomic data generated in this study was deposited to ProteomeXchange via the PRIDE database (PXD022482, PXD022553, PXD032031). Previously-published leukemia proteomic data (Nix et al, Cancer Discov, 2021) was downloaded from PRIDE (PXD016800). RNA sequencing data generated in this study was uploaded to GEO (GSE160572). Processed leukemia RNA sequencing data (Nix et al) was downloaded from GEO (GSE142447). Source data are provided with this paper. There are no restrictions on data availability.

Field-specific reporting

Please select the one below that is the best fit for your research. If you are not sure, read the appropriate sections before making your selection.

☒ Life sciences ☐ Behavioural & social sciences ☐ Ecological, evolutionary & environmental sciences

For a reference copy of the document with all sections, see nature.com/documents/nr-reporting-summary-flat.pdf

Life sciences study design

All studies must disclose on these points even when the disclosure is negative.

Sample size	All proteomic experiments on cell lines were performed in biological triplicate. All RNA-seq experiments on cell lines were performed in biological duplicate. Other experiments (flow cytometry, drug treatment, etc) on cell lines were performed in biological or technical triplicate or quadruplicate as noted in the Figure legends. For primary sample analysis there was no pre-determined sample size and no specific power analysis to determine the number of primary samples to be used.
Data exclusions	No data exclusions.
Replication	Multiple biological and/or technical replicates were performed for all experiments, and are specifically noted in the figure legends. We did not experience any inability to reproduce results.
Randomization	For resistant vs. wild-type experiments, proteomic samples were injected on the mass spectrometer using block randomization between parent and resistant cell lines to avoid batch effects.
Blinding	Blinding is not directly relevant to the study as all proteomic experimental data were analyzed simultaneously in MaxQuant with outputs directly used for downstream interpretation.

Reporting for specific materials, systems and methods

We require information from authors about some types of materials, experimental systems and methods used in many studies. Here, indicate whether each material, system or method listed is relevant to your study. If you are not sure if a list item applies to your research, read the appropriate section before selecting a response.

Materials & experimental systems

n/a	Involved in the study	
☐	☒	Antibodies
☐	☒	Eukaryotic cell lines
☒	☐	Palaeontology and archaeology
☒	☐	Animals and other organisms
☐	☒	Human research participants
☒	☐	Clinical data
☒	☐	Dual use research of concern

Methods

n/a	Involved in the study	
☒	☐	ChIP-seq
☐	☒	Flow cytometry
☒	☐	MRI-based neuroimaging

Antibodies

Antibodies used	We list here all antibodies used in this study as well as URL to manufacturer website describing associated validation procedure for each antibody. Validation information for antibodies used are available from manufacturer websites at the links provided here: FITC Mouse anti-human CD48 (BD Biosciences, clone TU145, https://www.fishersci.com/shop/products/anti-cd48-fitc-clone-tu145-bd/BDB555759), FITC Mouse anti-human CD38 (BD Biosciences, clone HIT2, https://www.bdbiosciences.com/en-us/products/reagents/flow-cytometry-reagents/research-reagents/single-color-antibodies-ruo/fitc-mouse-anti-human-cd38.560982), FITC Mouse IgG2b-k isotype control (https://www.fishersci.com/shop/products/anti-igg2b-mouse-fitc-clone-27-35-isotype-control-bd/BDB555742), CD138 (BD Biosciences, 562097, https://www.bdbiosciences.com/content/dam/bdb/products/global/reagents/flow-cytometry-reagents/research-reagents/single-color-antibodies-ruo/562097_base/pdf/562097.pdf, 552026, https://www.citeab.com/antibodies/2408174-552026-bd-pharmingen-pe-mouse-anti-human-cd138), BCMA (BioLegend 357504, https://www.biolegend.com/en-us/search-results/pe-anti-human-cd269-bcma-antibody-8446?GroupID=BLG11472), CD53 (BD Biosciences, 555508, https://www.bdbiosciences.com/ja-jp/products/reagents/flow-cytometry-reagents/research-reagents/single-color-antibodies-ruo/pe-mouse-anti-human-cd53.555508), CD10 (BD Biosciences, 561002, https://www.bdbiosciences.com/content/dam/bdb/products/global/reagents/flow-cytometry-

reagents/research-reagents/single-color-antibodies-ruo/555375_base/pdf/561002.pdf), CD151 (BD Biosciences, 556057, https://www.bdbiosciences.com/en-au/products/reagents/flow-cytometry-reagents/research-reagents/single-color-antibodies-ruo/pe-mouse-anti-human-cd151.556057), CD50/ICAM3 (BD Biosciences, 555958, https://www.bdbiosciences.com/en-au/products/reagents/flow-cytometry-reagents/research-reagents/single-color-antibodies-ruo/fitc-mouse-anti-human-cd50.555958), ITGB7 (BD Biosciences, 555945, https://www.citeab.com/antibodies/2410769-555945-bd-pharmingen-pe-rat-anti-integrin-7), MUC1/CD227 (BD Biosciences, 559774, https://www.bdbiosciences.com/en-au/products/reagents/flow-cytometry-reagents/research-reagents/single-color-antibodies-ruo/fitc-mouse-anti-human-muc1-cd227.559774), CCR10 (BD Biosciences, clone 1B5, https://www.bdbiosciences.com/en-eu/products/reagents/flow-cytometry-reagents/research-reagents/single-color-antibodies-ruo/bb515-mouse-anti-human-ccr10.564769), CD19 (BD, 555412, https://www.bdbiosciences.com/en-eu/products/reagents/flow-cytometry-reagents/research-reagents/single-color-antibodies-ruo/fitc-mouse-anti-human-cd19.555412; Biolegend 363036, https://www.biolegend.com/en-us/products/pacific-blue-anti-human-cd19-antibody-13773?GroupID=BLG7899), CD3 (Biolegend 344840, https://www.biolegend.com/ja-jp/products/apc-fire-750-anti-human-cd3-antibody-13004), IgG1 Mouse Control (Biolegend 400141, https://www.biolegend.com/en-us/search-results/apc-mouse-igg1-kappa-isotype-ctrl-icfc-3033; Biolegend 400196, https://www.biolegend.com/en-us/search-results/apc-fire-750-mouse-igg1-kappa-isotype-ctrl-13011?GroupID=BLG15288, BD Biosciences 555748, https://www.citeab.com/antibodies/10162097-555748-bd-pharmingen-fitc-mouse-igg1-isotype-co), IgG2a Mouse Control (BD 555576), https://www.bdbiosciences.com/en-eu/products/reagents/flow-cytometry-reagents/research-reagents/flow-cytometry-controls-and-lysates/apc-mouse-igg2a-isotype-control.555576, c-Myc, Biotin (Milteny Biotech 130-124-877), https://www.miltenyibiotec.com/US-en/products/c-myc-antibody-anti-human-mouse-rat-sh1-26e7-1-3.html#gref, Biotin-SP (long spacer) AffiniPure Goat Anti-Mouse IgG, F(ab') fragment specific (Jackson Labs, 115-065-072), Fluorescein $_2$ (FITC) AffiniPure F(ab') Fragment Goat Anti-Human IgG (Jackson Labs, 109-096-098), $_2$ https://www.jacksonimmuno.com/catalog/products/115-065-072.

Validation

See above.

Eukaryotic cell lines

Policy information about cell lines

Cell line source(s)

RS4;11 and MM.1S cells were obtained from ATCC (cat# CRL-1873, CRL-2974). AMO-1, KMS12-PE, and RPMI-8226 were obtained from DSMZ (cat# ACC-538, ACC-606, ACC-402). Drug resistant cell lines and paired WT lines, including Bortezomib and Carfilzomib resistant cells derived from AMO-1, L363, RPMI-8226, and ARH-77, and Lenalidomide resistant cells derived from H929 and OPM-2 are described in previous publications (Soriano, G. P. et al. Leukemia 30, 2198–2207 (2016) for proteosome-inhibitor resistant cells; Lopez-Girona, A. et al. Leukemia 26, 2326–2335 (2012) for lenalidomide-resistant cells).

Authentication

Cell lines were authenticated using STR genotyping from ATCC

Mycoplasma contamination

Cell lines tested negative for mycoplasma contamination in routine testing.

Commonly misidentified lines
(See ICLAC register)

No commonly misidentified cell lines were used in this study.

Human research participants

Policy information about studies involving human research participants

Population characteristics	Population characteristics are described in Supplementary Table 2, including patient sex; age; clinical diagnosis; prior treatment or no prior treatment; FISH genotyping results on tumor cells. These characteristics are approved for extraction for research per UCSF IRB-approved protocols (see Ethics Oversight, below).
Recruitment	Patients enrolling in the Multiple Myeloma Translational Initiative protocol are recruited by clinical investigators in the UCSF Dept. of Hematology/Oncology and are all formally listed and approved on the associated IRB approval. No compensation is provided for research participants under this protocol. For bone marrow core biopsies obtained under the UCSF Hematopathology protocol, no recruitment is performed as all FFPE-embedded tissues were obtained 6 months or more in the past and are previously housed within the UCSF Hematopathology archive.
Ethics oversight	All human samples were obtained in accordance with the Declaration of Helsinki and through protocols approved by the UCSF Committee on Human Research Institutional Review Board (IRB). These include all primary myeloma bone marrow aspirate specimens obtained through the UCSF Multiple Myeloma Translational Initiative (MMTI) protocol (UCSF IRB approval 10-00545) and bone marrow core biopsies obtained through the UCSF General Hematopathology Protocol (Hemepath) (UCSF IRB approval 10-01080). All patients participating on the MMTI protocol complete and sign informed consent prior to data or biospecimen collection. For the retrospective Hemepath protocol, the UCSF IRB has waived the need to obtain informed consent prior to extracting data for research due to: “Many subjects are no longer being followed at the institution or are deceased”; “The attempt to contact subjects poses a greater risk than this study”; and “The large number of records required makes it impracticable to contact all potential subjects.”

Note that full information on the approval of the study protocol must also be provided in the manuscript.

Flow Cytometry

Plots

Confirm that:

- [x] The axis labels state the marker and fluorochrome used (e.g. CD4-FITC).
- [x] The axis scales are clearly visible. Include numbers along axes only for bottom left plot of group (a 'group' is an analysis of identical markers).
- [x] All plots are contour plots with outliers or pseudocolor plots.
- [x] A numerical value for number of cells or percentage (with statistics) is provided.

Methodology

Sample preparation	Cells were resuspended in FACS buffer (5% FBS in D-PBS) and stained with antibodies for 1-2 hours on ice, washed with FACS buffer, and then resuspended in FACS buffer. For experiments where live cells populations were studied, cells were resuspended in FACS buffer. Samples were analyzed using either a Cytoflex Flow Cytometer (Beckman Coulter) or FACSAria II flow cytometer (BD Biosciences). Data Analysis was done using FlowJo software, v10.8.1 or v8.8.1. For analyses including live/dead stains, cells were resuspended in FACS buffer containing Sytox Green or Sytox Red (Thermo). For drug treatment experiments, cells were plated into 96 well plates and treated for 48 hours with compounds unless otherwise noted. For quantitative flow cytometry, antibodies used were FITC Mouse anti-human CD48 (BD Biosciences, clone TU145), FITC Mouse anti-human CD38 (BD Biosciences, clone HIT2), FITC Mouse IgG1k isotype control (BD Biosciences, clone 27-35), and unstained respectively. using calibrated beads (Bangs Labortories), 200 µL of FACS buffer + 1 drop of beads per well (one wells for “blank” beads, and other for FITC-Beads). Compensation was performed using UltraComp eBeads™, compensation Beads (Invitrogen). Calibration curve for quantification beads were performed on the Quantitative software QuickCal v2.3 (Bangs Laboratories, Inc.) before FITC analysis on multiple myeloma cell lines. Additional antibodies used are as follows: CD138 (BD Biosciences, 562097, 552026), BCMA (BD Biosciences, 552026, BioLegend 357504), CD53 (BD Biosciences, 555508), CD10 (BD Biosciences, 561002), CD151 (BD Biosciences, 556057), CD50/ICAM3 (BD Biosciences, 555958), ITGB7 (BD Biosciences, 555945), MUC1/CD227 (BD Biosciences, 559774), CCR10 (BD Biosciences, clone 1B5), CD19 (BD, 555412; Biolegend 363036), CD3 (Biolegend 344840), IgG1 Mouse Control (Biolegend 400141; Biolegend 400196, BD Biosciences 555748), IgG2a Mouse Control (BD 555576), c-Myc, Biotin (Milteny Biotech 130-124-877), Biotin-SP (long spacer) AffiniPure Goat Anti-Mouse IgG, F(ab')$_2$ fragment specific (Jackson Labs, 115-065-072), Fluorescein (FITC) AffiniPure F(ab')$_2$ Fragment Goat Anti-Human IgG (Jackson Labs, 109-096-098). Additional isotype antibodies were ordered from BD Biosciences and BioLegend as per manufacturer recommendations.
Instrument	BC Cytoflex was used for all analytical flow cytometry.

Software	FlowJo versions 8.8.6 and 10.8 were used.
Cell population abundance	For cell line analysis, cell population was gated on all live, singlet cells. For primary sample analysis, myeloma tumor cells were assessed based on live, singlet cells exhibiting the marker pattern of CD138+/CD19- and were assessed for either CD38, CD48, CCR10, or MUC-1. These cells were quantified for abundance of the population of interest.
Gating strategy	As described in Supp. Fig. 2C, gating on primary tumor cells was accomplished based on cells in the lymphocyte gate (low-SSC, moderate FSC) that were first gated on CD138+/CD19-, then confirmed to be CD38+. These cells were assessed for other markers (CCR10, CD48, MUC-1) as described. As described in Supp. Fig. 2D, FSC and SSC patterns were used to distinguish presumed monocytic, neutrophilic, and lymphocyte populations, followed by CD14, CD45, and CD3 staining as described.

☒ Tick this box to confirm that a figure exemplifying the gating strategy is provided in the Supplementary Information.
